# 1-Oxo-2,2,6,6-tetramethylpiperidinium bromide converts α-H *N,N-*dialkylhydroxylamines to nitrones via a two-electron oxidation mechanism

**DOI:** 10.1038/s41598-018-33639-w

**Published:** 2018-10-17

**Authors:** Anastas D. Stoyanovsky, Detcho A. Stoyanovsky

**Affiliations:** 1 IBM Watson, Pittsburgh, Pennsylvania USA; 2 Department of Environmental and Occupational Health, University of Pittsburgh, Pittsburgh, Pennsylvania USA

## Abstract

Herein we provide experimental proof that 1-oxo-2,2,6,6-tetramethylpiperidinium bromide converts α-H *N*,*N*-dialkylhydroxylamines to nitrones via a two-electron oxidation mechanism. The reactions reported are rapid, proceed under mild conditions, and afford nitrones in excellent yields.

## Introduction

**Figure 1:**
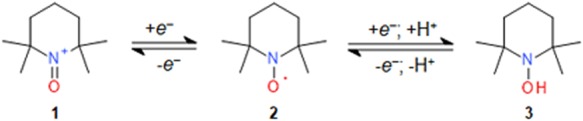
Redox equilibria between the oxoammonium, nitroxide and hydroxylamine forms of TEMPO.

Nitroxides (aminoxyl radicals) have attracted considerable interest as catalysts in (bio)organic reactions^1–5^, electron paramagnetic resonance (EPR) probes^6,7^, and drugs that mitigate oxidative injury^8–10^. The stability of most nitroxides containing tertiary α- and α′- carbon atoms relative to their >N-O^•^ group is sufficiently high to allow their isolation as pure compounds (Fig. 1, **2**- 2,2,6,6-tetramethylpiperidine 1-oxyl; TEMPO). In solutions, however, **2** readily undergoes redox interconversion to oxoammonium cation (**1**) and hydroxylamine (**3**).

Halides of **1** can be synthesized by oxidation of **2** with I_2_, Br_2_ and Cl_2_ (**2** + 1/2X_2_ → 1.X) or by acid-catalyzed disproportionation of **2** (HCl + **2** + **2** → **1**.**Cl** + **3**)^11–14^, while in biological systems reduction of oxygen-centered radicals by **2** affords **1**^3,4^. Oxoammonium salts are versatile oxidants that interconvert a number of functional groups. Their reactivity is illustrated by the conversion of primary and secondary alcohols to aldehydes and ketones, respectively, enolizable ketones to diketones, and *N*,*N-*dialkylamines to alkylamines^2,14–20^. Most reactions of **1** proceed with formation of **2** and/or **3** as end-reaction products, thus allowing their performance under catalytic conditions where a terminal oxidant shifts the equilibria between **1**, **2**, and **3** in favor of **1**^21^.

**Figure 2:**
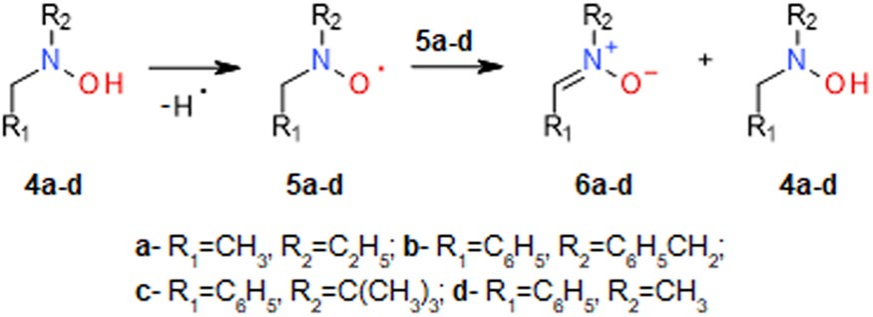
Disproportionation of α-H nitroxides to nitrones and hydroxylamines.

While it has been shown that in neutral solutions **1** oxidizes hydroxylamine **3** to nitroxide **2** via electron transfer^22,23^, reactions of **1** with *N,N*-dialkylhydroxylamines containing an α-H atom have not been studied thus far. One-electron oxidation of α-H *N*,*N*-dialkylhydroxylamines yields nitroxides which exhibit half-lives ranging from seconds to hours. The low stability of these radicals has been associated with their disproportionation to the parent hydroxylamines and nitrones (Fig. 2)^24–26^, and, competitively, to N-C bond cleavage^27–29^. Herein, we report that **1** readily converts a series of α-H *N*,*N*-dialkylhydroxylamines to nitrones via a two-electron oxidation mechanism.

## Results and Discussion

### EPR and HPLC-UV analysis of the oxidation of *N*,*N*-dialkylhydroxylamines by **1.Br**

**Figure 3:**
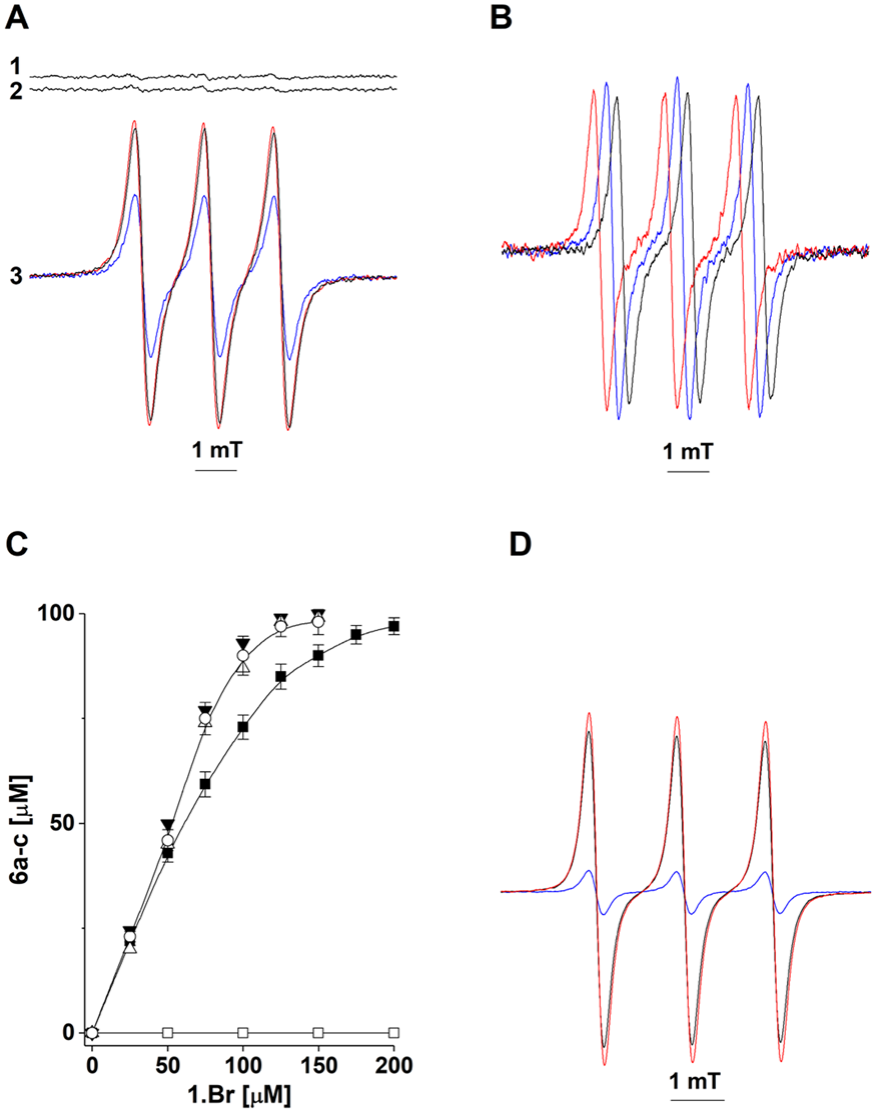
EPR- and HPLC-monitored oxidation of hydroxylamines **4a-c** to nitroxides **5a-c** and nitrones **6a-c**. Reactions were carried out at 25 °C in CH_3_CN containing 10% water. A1,2- EPR spectra of **1**.**Br** (50 μM) and **3** (150 μM); traces 3- **1**.**Br** (blue and black spectra, 25 μM and 50 μM, respectively) plus hydroxylamine **3** (150 μM); red trace- 100 μM standard solution of nitroxide **2**. B- EPR spectra of **4a-c** (100 μM) plus **1**.**Br** (25 μM); shifted spectra are presented to show that in all reactions comparable amounts of nitroxide **2** were formed. C- HPLC-monitored formation of nitrones **6a-c** in reaction solutions consisting of **1**.**Br** and **4a** (100 μM; filled triangles), **4b** (100 μM; open circles), and **4c** (100 μM; open triangles); filled rectangles, **1**.**Br** plus **4c** (100 μM) in ethanol. Open rectangles, **4b** in the absence of **1** (incubation time, 20 min). The data are presented as mean values of three independent experiments ± the standard error. D- EPR spectra of **1**.**Br** (125 μM) plus **4c** (100 μM) before (blue trace) and after (black trace) addition of C_2_H_5_ONa (30 mM; red trace- 100 μM standard solution of nitroxide **2**).

The EPR spectra presented in Fig. 3A show the oxidation of hydroxylamine **3** (150 μM) by **1**. In CH_3_CN containing either **3** or **1.Br** (50 μM), we did not observe well resolved EPR spectra (traces 1 and 2). Addition of **1.Br** (Fig. 3A3; blue and black tracing, 25 μM and 50 μM, respectively) to a solution of **3** led to the formation of **2**, as assessed by the appearance of the typical 3-line EPR spectrum of this nitroxide (a_N_ = 1.715 mT). Following one-electron oxidation, in this reaction one molecule of **1** reacted with **3** to generate two molecules of nitroxide **2** (red tracing, 100 μM standard solution of **2**). Under these experimental conditions, the oxidation of **3** was too fast to record its kinetic profile by conventional EPR spectrometry. Similarly, the oxidation of the α-H hydroxylamines **4a–c** (100 μM) by **1.Br** (25 μM) proceeded with concomitant formation of nitroxide **2** (Fig. 3B, blue, red and black spectrum, respectively). As suggested by the constant magnitude of these spectra, each reaction was completed in less than 30 seconds, which is the approximate time required for sample preparation and data acquisition.

HPLC analysis of the reaction solutions revealed that the oxidation of **4a–c** by **1** also led to formation of nitrones **6a–c** (Fig. 3C; Supplementary Information; SI). The requirement for a slight stoichiometric excess of the oxoammonium salt for complete oxidation of **4a-c** most likely reflected the competition between **4a–c** as reactants and the end-reaction product **3** for **1**. In support of this assumption, the oxidation of hydroxylamine **4b** (100 μM) by **1** (125 μM) to nitrone **6b** (98 μM; Fig. 3C) was paralleled by formation of both nitroxide **2** (14 μM; Fig. 3D, blue tracing) and hydroxylamine **3** (79 μM; Fig. 3D, black tracing). Quantification of **3** was performed after its oxidation to **2** in alkaline milieu (>N-O^−^ + O_2_ → >N-O^●^ + O_2_^●−^ ^25^; red tracing, 100 μM standard solution of **2**). In the absence of oxoammonium salt, hydroxylamine **4b** did not oxidize to nitrone **6b** (Fig. 3C, open rectangles).

Similar distribution of the products was observed when reactions were carried out with **4a-c** in CH_3_OH, C_2_H_5_OH, CH_3_CN, and CH_2_Cl_2_ with the notion that the oxoammonium cation **1** does not react with CH_3_CN and CH_2_Cl_2_ to any significant extent but does oxidize primary alcohols to aldehydes. Hence, in ethanol, the complete oxidation of hydroxylamine **4c** to nitrone **6c** required larger excess of **1** (Fig. 3C, filled rectangles). Ethanol, however, did not prevent the formation of **6c**, which suggests that the formation of nitrones would be the preponderant process in polyfunctional compounds containing both OH and NOH groups. The stoichiometry of the reactions further suggests that nitrones were not formed via the intermediate formation and disproportionation of α-H nitroxides as completion of the latter process would require 2 molar equivalents of **1** for the oxidation of the hydroxylamines (Fig. 2). This conclusion was further supported by kinetic analyses of the decay of nitroxides **5b**,**c**.

**Figure 4:**
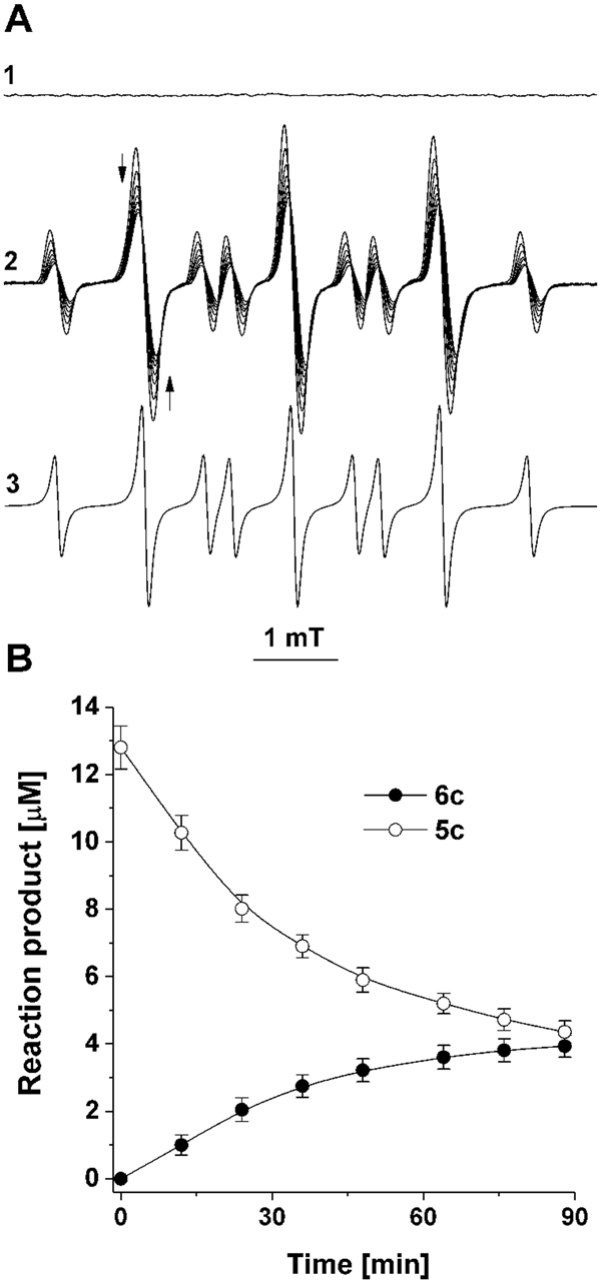
EPR analysis of the decay of nitroxide **5c** and HPLC-monitored formation of nitrone **6c**. A1- EPR spectrum of **4c** (200 μM). A2- **4c** plus NaOH (0.5 M). After incubation for 4 min, the reaction solution was titrated with acetic acid to pH 6.0 and consecutive EPR spectra were recorded with time intervals of 12 min. Arrows indicate the directions of the spectral changes. A3- computer simulation of the EPR spectrum of nitroxide **5c** (simulation parameters: a_H_ = 1.03 mT; a_N_ = 1.76 mT; number of equivalent protons, 2). B- EPR (open circles)- and HPLC (filled circle)-monitored changes in the concentrations of nitroxide **5c** and nitrone **6c**. The data are presented as mean values of three independent experiments ± the standard error.

In Fig. 4A1 is shown the EPR spectrum of water containing ethanol (25%), ethylenediaminetetraacetic acid (EDTA; 100 μM), and hydroxylamine **4c** (200 μM). Addition of NaOH (0.5 M) led to the appearance of the EPR spectrum of nitroxide **5c** (in mT, a_H_ = 1.0318; a_N_ = 1.7607), which reflected the oxidation of the aminoxyl anion of **4c** by oxygen^25^ (Fig. 4A2; spectrum 3, computer simulation of the EPR spectrum of **5c**). We then acidified the reaction solution to pH 6.0 with acetic acid and recorded both the decreases in the EPR spectrum of **5c** (Figs 2B and 4A2, open circles; the spin concentration of **5c** was determined by double integration of the EPR signals using authentic **2** as a standard) and the formation of nitrone **6c** (as assessed by HPLC; Fig. 4B, filled circles). In agreement with Fig. 2, two molecules of **5c** were consumed for each molecule of **6c** formed in the reaction. Importantly, the half-life of **5c** (t_1/2_ ~ 60 minutes) largely exceeded the time required for oxidation of **4c** by **1**, thus excluding the generation and disproportionation of nitroxide **5c** as a reaction mechanism responsible for the formation of nitrone **6c**.

We further carried out experiments to verify whether nitroxide **5b** is formed during the oxidation of hydroxylamine **4b** by **1**, with the expectation that the large difference in the hyperfine splitting constants of **2** and **5b**^25^ will allow their simultaneous EPR detection in the reaction milieu. In Fig. 5.1 is shown the EPR spectrum of **5b** generated in an alkaline solution of **4b** (in mT, a_H_ = 1.097; a_N_ = 1.761; spectrum 3, computer simulation of the EPR spectrum of **5b**). In agreement with the data reported in ref.^25^, alkalization of the solution of **4b** led to the appearance of the EPR spectrum of **5b**, which increased for ~1 min and then remained constant for ~30 min. Acidification of the reaction solution to pH 6.0 and following kinetic analysis of the decay **5b** established that the half-life of this radical is ~4 min (data not shown), which provides ample time for its EPR analysis. In Fig. 5.3 is presented the EPR spectrum of a reaction solution consisting of hydroxylamine **4b** (1 mM) and **1**.**Br** (1 mM), which contains as a major component the three spectral lines of **2** (red tracing). By comparing the latter spectrum with that of **5b** as a standard (4.9 μM; blue tracing), we observed that **5b** was present in the reaction solution at a submicromolar concentration, or less than 0.1% of the expected (~0.8 mM) for one-electron oxidation of **4b**; in Fig. 5.3, the first two spectral lines of **5b** are denoted with arrows.

**Figure 5:**
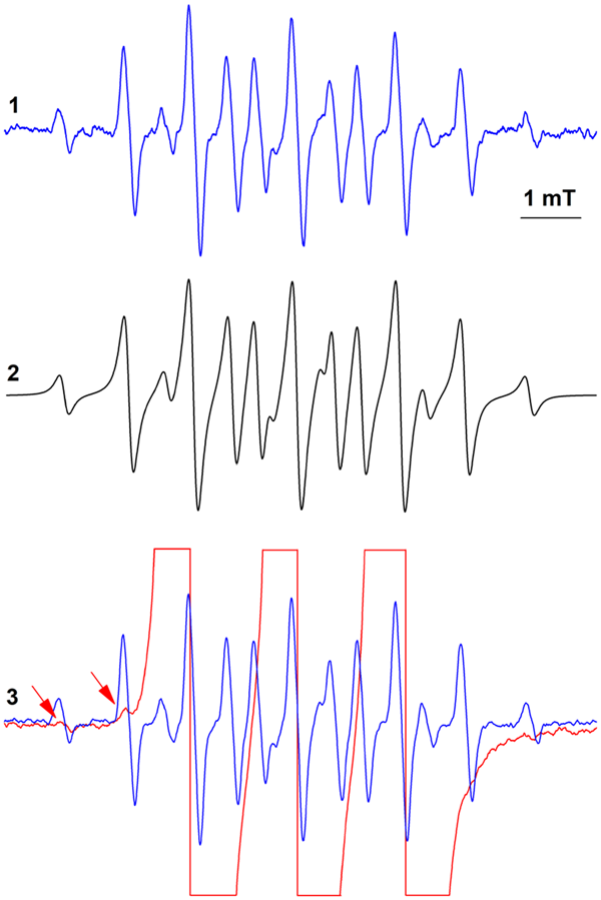
EPR analysis of the oxidation of hydroxylamine **4b** by **1.Br**. Reactions were carried out at room temperature in 25% methanol. Spectrum 1- **4b** (200 μM) plus NaOH (0.5 M). Spectrum 2- Computer simulation of the EPR spectrum of nitroxide **5b** (simulation parameters: a_H_ = 1.11 mT; a_N_ = 1.76 mT; number of equivalent protons, 4). Traces 3- Overlapped spectra of **5a** (1 mM) plus **1.Br** (1 mM; red tracing) and **5a** (200 μM) plus NaOH (05 M; blue tracing).

**Figure 6:**
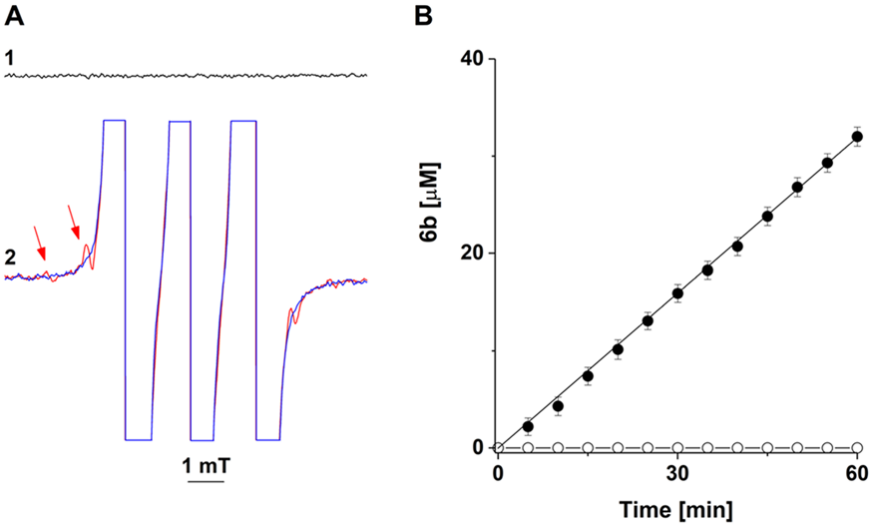
EPR (**A**)- and HPLC (**B**)-monitored oxidation of **4b** by **2** to nitroxide 5b (**A**) and nitrone **6b** (**B**). Reactions were carried out at room temperature in 25% methanol. A1- **4b** (1 mM). A2, blue tracing- 2 (1 mM). A2, red tracing- **4b** + **2**; the first two lines in the EPR spectrum of **5b** are denoted with red arrows. B- Formation of nitrone **6b** in a solution of hydroxylamine **4b** (1 mM) in the absence (open circles) and the presence (filled circles) of nitroxide **2** (1 mM). HPLC separations were performed as indicated in SI. The data are presented as mean values of three independent experiments ± the standard error.

The data presented in Fig. 6A indicate that the trace amounts of nitroxide **5b** were formed via a secondary reaction in which the end reaction product **2** oxidized the parent hydroxylamine **4b** (Fig. 6A; **4b** + **2** → **5b** + **3**); the EPR spectrum of **5b** (~1 μM) could be observed upon addition of nitroxide **2** (1 mM) to a solution of hydroxylamine **4b** (1 mM; Fig. 6A, red tracing). Incubation of this reaction solution led to a slow formation of nitrone **6b** (Fig. 6B; yield of nitrone for 1 hour, 3%), presumably via disproportionation of **5b**. While under these experimental conditions reaction **4b** + **1** → **6b** was completed in less than 1 minute (Fig. 1C), the data presented in Fig. 6B indicate that, when the oxoammonium salt **1** was used as an oxidant, the secondary oxidation of the hydroxylamine did not significantly contribute to the formation of nitrone **6b**.

**Figure 7:**
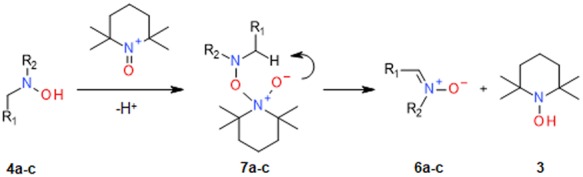
Proposed mechanism for the oxidation of α-H *N*,*N*-dialkylhydroxylamines to nitrones by 1.

Altogether, the data obtained support a two-electron oxidation mechanism for the reaction between the α-H *N*,*N-*dialkylhydroxylamines and **1**, which is reminiscent of the oxidation of alcohols by oxoammonium salts^20^ (Fig. 7). Accordingly, the stoichiometric oxidation of **4a-c** by **1** is proposed to proceed via formation of reaction intermediates **7a-c** with concomitant cyclic elimination of the end-reaction products **6a-c** and **3**.

### Oxidation of α-H *N*,*N*-dialkylhydroxylamines by **1.Br** under preparative conditions

Nitrones are widely used as reagents in reactions of cycloaddition and alkylation with organometalics, and as EPR spin-trapping probes. Hence, considerable research effort has been directed toward the synthesis of this class of compounds. The oxidation of α-H *N*,*N*-dialkylhydroxylamines has proven a principal method for the synthesis of nitrones, where HgO^30^, Ag_2_O^31^, MnO_2_^32^, hypervalent iodine reagents^33^, and copper complexes^34^ have been successfully used as oxidants. An alternative that uses nontoxic reagents has been reported by Alderson *et al*.^35^ and Cicchi *et al*.^36^ in which NaBrO and NaClO oxidize α-H *N*,*N-*dialkylhydroxylamines to nitrones with reaction times and yields ranging from 1 to 20 hours and 40% to 95%, respectively.

**Figure 8:**
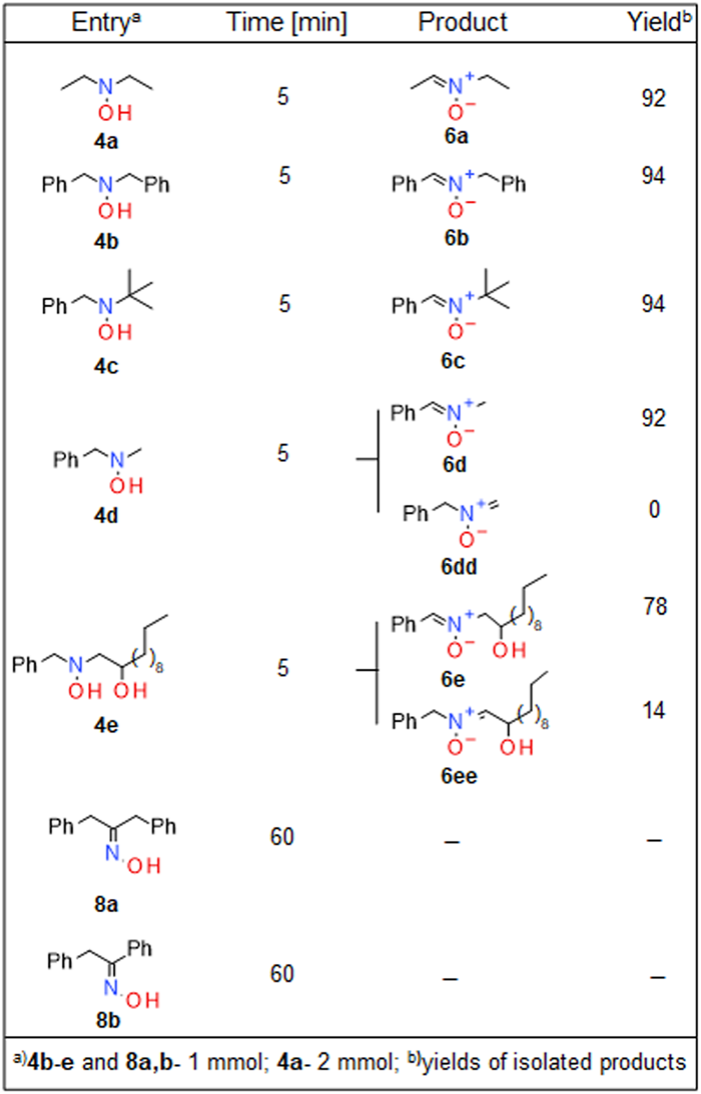
Oxidation of α-*N*,*N*-dialkylamines by **1.br** under preparative conditions.

The high rates of oxidation of α-H *N*,*N-*dialkylhydroxyl-amines by **1** and the excellent yields of nitrones under analytical conditions prompted us to scale-up the reactions to preparative amounts of hydroxylamines. At ambient temperature, 1–2 mmoles of **4a-e** (dissolved in 90% methanol) were efficiently oxidized by **1**.**Br** (Fig. 8). Hydroxylamines **4a**,**b**,**d** are standard substrates of oxidation and provide a foundation for comparison of different synthetic protocols; depending on the oxidant used, reactions with **4a**,**b**,**d** have been reported to proceed for hours and to afford nitrones with good to excellent yields^33,34,36,37^. In agreement with the data presented in Fig. 3C, maximal yields of nitrones were obtained with the use of 1.2 molar equivalents of **1**.**Br** per mole of hydroxylamine. In the absence of NaHCO_3_ the reactions proceeded with concomitant hydrolysis of the nitrones by HBr to aldehydes and *N-*alkylamines (data not shown). In the oxidation of **4d**, formation of **6dd** was not detected, whereas **4e** afforded regioisomers **6e** (yield, 78%) and **6ee** (yield, 14%; SI). Notably, **1** selectively oxidized the NOH of **4e** and not its OH group. Under these experimental conditions, oximes **8a**,**b** did not react with **1** to any significant extent, indicating that the reaction is specific for hydroxylamines and that deprotonation of the NOH group did not promote its oxidation, presumably via preferential addition of the corresponding aminoxyl anions to **1**. As estimated with MarvinSketch (ChemAxon; Cambridge, MA), in aqueous solutions the pKa values of the NOH group of **4b**, **8a** and **8b** are 15.57, 9.84 and 7.37, respectively.

## Conclusions

The data presented herein expand the list of functional groups that can be interconverted by oxoammonium salts. We show that **1** converts the >N-OH group into a nitrone group via a two-electron oxidation mechanism in each of a series of α-H dialkylhydroxylamines. The reaction is rapid, proceeds under mild conditions, and affords nitrones in excellent yields.

The interconversion between nitrones and hydroxylamines is a viable strategy for carbon-carbon formation that, when coupled with acidic hydrolysis of nitrone derivatives, can be applied to structural diversification of aldehydes (R_1_CH = O + R_2_NHOH → R_1_CH = N^+^(O^−^)R_2_ → R_1_R_3_CHN(OH)R_2_ → R_1_R_3_C = N^+^(O^−^)R_2_ → R_1_R_3_CO)^38–43^. To this end, the rapid oxidation of α-H *N*,*N*-dialkylhydroxylamines by oxoammonium salts may prove advantageous to the optimization of one-pot synthetic protocols. Since dealkylation of α-H *N*,*N*-dialkylhydroxylamines may also be of interest, the oxidation of this class of compounds by **1.Br** to nitrones can be followed by acid-catalyzed hydrolysis of the latter, which would afford aldehydes (or ketones) and *N*-alkylhydroxylamines.

## Materials and Methods

### Reagents

Hydroxylamines **4a,b** were purchased from TCI America, Inc. (Montgomeryville, PA). All other chemicals, including nitrones **6b**,**c**,**d** were purchased from Sigma (St. Louis, MO). Nitrone **6a** was obtained via oxidation of **4a** with Ag_2_O as reported in ref.^31^. Nitrones **6a-d** were used as external reference HPLC standards. Protocols for preparation of **1**.**Br**, **3** and **8a**,**e** are included in SI, along with NMR, HRMS, and HPLC data.

### General procedure for the oxidation of α-H *N*,*N*-dialkylhydroxylamines by **1.Br**

To a stirred suspension of α-H *N*,*N-*dialkylhydroxylamine (1 mmol) in methanol containing 10% water (v/v; 10 mL; 25 °C) and NaHCO_3_ (250 mg; 3 mmol) was added dropwise 1.25 equiv. of **1**.**Br** (dissolved in 10 mL CH_3_CN) over 5 min. Instant decolourisation of the dark-brown solution of **1**.**Br** followed the addition without any apparent effervescence. The inorganic salts were filtered off, washed with absolute ethanol (2 × 5 mL), and the solvents from the filtrate were rotor-evaporated (35 °C; 20 Torr). Nitrones from the dry residues were separated by column chromatography as indicated SI.

## Electronic supplementary material

Supplementary Information
