# Supplementary material for: 1-Oxo-2,2,6,6-tetramethylpiperidinium bromide converts α-H N,N-dialkylhydroxylamines to nitrones via a two-electron oxidation mechanism
Source: Sci Rep. 2018 Oct 17;8:15323. doi: 10.1038/s41598-018-33639-w (PMC6193029; doi:10.1038/s41598-018-33639-w)
Supplement: Supplementary file 1 — Supplementary Information [file 41598_2018_33639_MOESM1_ESM.pdf]

1-Oxo-2,2,6,6-tetramethylpiperidinium bromide converts α-H *N,N*-dialkylhydroxylamines to nitrones via a two-electron oxidation mechanism

Anastas D. Stoyanovsky[1] and Detcho A. Stoyanovsky[2]*

Supplementary Information

Table of Contents

Material and Methods	Page 2
Synthetic protocols	Page 2
NMR and HRMS data	Page 3
Copies ^{1}H and ^{13}C NMR spectra **4b-e**, **6a-d**, and **8ab**	Page 4
HPLC separation of **6e** and **6ee**	Page 12
HRMS, MS/MS, and NMR analysis of **6ee** and **6e**	Page 13
Copies of HRMS spectra	Page 16
HPLC-UV analysis of **4a-d**	Page 18
References	Page 18

Materials: Hydroxylamines **4a,b** have been purchased from TCI America, Inc. (Montgomeryville, PA). All other chemicals, including nitrones **6b,c,d** and Si-TEMPO have been purchased from Sigma (St. Louis, MO). Nitrone **6a** has been obtained via oxidation of **4a** with Ag_2O as reported in ref [1]; nitrones **6a-d** have been used as external reference HPLC standards.

Equipment and settings: *EPR spectra* were recorded at room temperature using a JEOL-RE1X spectrometer (Kyoto, Japan). Spectrometer settings were: field center 335.094 mT, microwave power 10 mW, sweep time 120 seconds, time constant 0.1 s, modulation width 0.1 mT, and amplification of 100. EPR spectra simulations were performed with a JEOL computer program.

NMR spectra were recorded with a Bruker 500 MHz Wide Bore spectrometer. Analytes were dissolved in $CDCl_3$.

Mass spectral analysis was performed with Shimadzu Instrument LCMS-2020. Analytes were dissolved in CH_2Cl_2 containing 1% oxalic acid.

Analytical HPLC separations of nitrones were performed on a C18 reverse phase column (Luna Omega Polar, 5 μm particle size; 4.6 x 250 mm; Phenomenex. Nitrones were eluted with a mobile phase consisting of methanol containing either 30% (**6b-d**) or 10% (**6e** and **6ee**) water, or water containing 20% methanol (**6a**). Analytes were detected with a Shimadzu SPD-M10Avp photodiode detector (λ_{max}(**6a-d, e,ee**) = 222, 291, 289, 287, 290, and 236 nm, respectively).

Preparative HPLC separations of nitrones **6a-d** and precursor hydroxylamines were performed on Silica column (Asentis Si; 10 x 250 mm; 5μm particle size; Supelco, Inc.; Bellefonte, PA). Elutions were carried out with a Shimadzu LC8A preparative HPLC pump and mobile phase of hexane containing either ethyl acetate (10 - 30%; **6b-d**) or diethyl ether (5%; **4c,d,e**). Nitrone **6a** was eluted with CH_2Cl_2 containing 2% methanol. Nitrones **6e** and **6ee** were separated on a C18 matrix (column, 2.12 x 25 cm; particle size, 5 μm; Supelco, Inc.; Bellefonte, PA),

Software: NMR spectra were analyzed with MestReNova (Mestrelab Research, S.L.; Escondido, CA, USA). HPLC data were acquired with EZchrome 4.2 (Agilent Technologies; Santa Clara, CA, USA). Figures were prepared with OriginLab v7.0 (Northampton, MA, USA).

Synthesis: 1-hydroxy-2,2,6,6-tetramethyl-piperidine (**3**) was obtained via reduction of **2** (50 mg; 0.32 mmol) dissolved in acetyl acetate (3 mL) with ascorbic acid (0.3 g; 1.7 mmol) dissolved in H_2O (2 mL). The bi-phasic reaction system was vigorously mixed for 10 min, which led to discoloration of the initially dark-red solution of **2** with concomitant disappearance of the EPR spectrum of the nitroxide. Thereafter, the organic phase was removed and the water phase extracted with ethyl acetate (4 x 2 mL). The unified extracts were mixed with 100 mL of helium-deaerated absolute ethanol and the solvents were evaporated under vacuum (25 °C; 5 torr) to afford **3** as viscous colorless oil (48 mg; yield, 95%). When kept at -20 °C in helium-deaerated hexane for up to 1 month, **3** did not decompose to any significant extent. In the presence of oxygen, however, **3** slowly oxidized to nitroxide **2**. Quantification of **3** (10 – 100 μM) was performed by EPR spectrometry after its oxidation in ethanol to nitroxide **2** upon addition of sodium ethoxide (30 mM). EPR spectra were recorded after initially purging the reaction solutions with air (1 min) and then with helium (5 min).

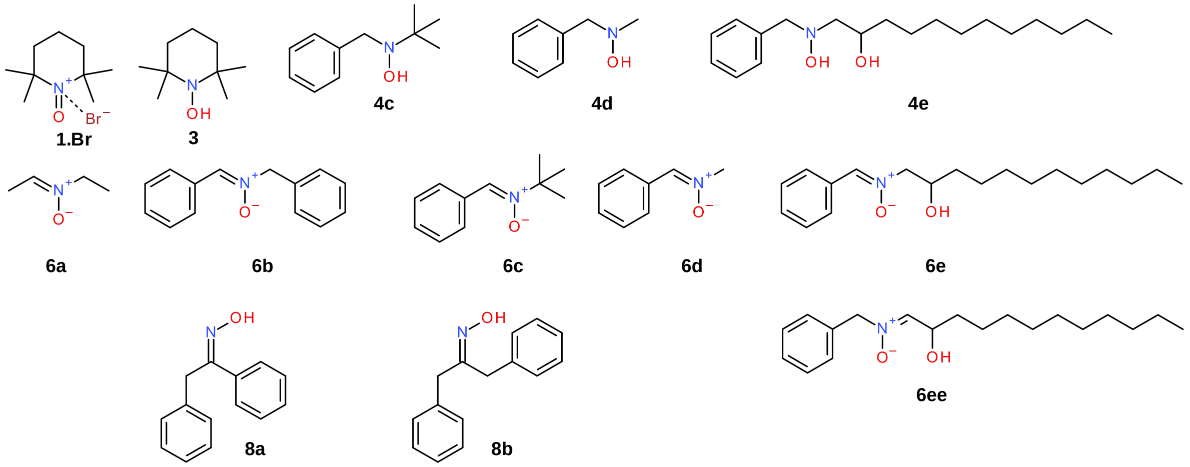

Oximes **8a,b** were prepared as reported in ref. [2]. Hydroxylamines **4c,d** have been synthesized via reduction of nitrones **6c,d** with $NaBH_4$ as reported in ref. [3], while **4e** has been prepared by addition of *N*-benzylhydroxylamine to 1,2-epoxydodecane as described in ref. [4]. 2,2,6,6-Tetramethylpiperidine-1-oxoammonium bromide (**1.Br**) has been synthesized via oxidation of nitroxide **2** with Br_2 as described in ref. [5]. Nitrones **6a – 6ee** were synthesized via oxidation of hydroxylamines **4a-e** with **1.Br** as described in the General Procedure section of the manuscript. Quantitative EPR measurements of the redox interconversion between **1.Br**, nitroxide **2** and hydroxylamine **3** are presented in Figure 1 in the manuscript. Hydroxylamines **4c-e** and nitrones **6a-ee** have been purified by HPLC as indicated in Methods and analyzed by NMR and HRMS.

^{1}H-NMR analysis

4c: δH(500 MHz; $CDCl_3$; Me_4Si) 1.21 (9 H, s, Me), 3.78 (2 H, s, CH_2), 4.46 (1 H, br s, NOH), 7.23-7.41 (6 H, m, CH, Ph).

4d: δH(500 MHz; $CDCl_3$; Me_4Si) 1.52 (3 H, s, Me), 3.71 (2 H, s, CH_2), 7.22-7.37 (5 H, m, Ph).

4e: δH(500 MHz; $CDCl_3$; Me_4Si) 0.93(3 H, t, Me), 1.11 – 1.51 (18 H, m, CH_2), 2.45 – 2.8 (2 H, m, CH_2), 3.7 – 3.9 (3 H, m, CH, CH_2), 7.2-7.4 (5H, m, Ph).

6a: δH(500 MHz; $CDCl_3$; Me_4Si) 1.34 (3 H, t, Me), 1.88 (3 H, d, Me), 3.69 (2 H, q, CH_2), 76.72 (1 H, m, CH).

6b: δH(500 MHz; $CDCl_3$; Me_4Si) 5.04 (2 H, s, CH_2), 7.25 – 7.55 (9 H, m, CH, Ph), 8.18 – 8.24 (2 H, m, Ph).

6c: δH(500 MHz; $CDCl_3$; Me_4Si) 1.59 (9 H, s, Me), 7.31 – 7.45 (3 H, m, Ph), 7.52 (1 H, s, CH), 8.28 (2 H, m, Ph).

6d: δH(500 MHz; $CDCl_3$; Me_4Si) 3.81 (3 H, s, Me), 7.25 – 7.39 (4 H, m, CH, Ph), 8.05 – 8.25 (2 H, m, Ph).

6e: δH(500 MHz; $CDCl_3$; Me_4Si) 0.75 (3 H, t, Me), 0.85 – 1.75 (18 H, m, CH_2), 3.6 – 3.95 (2 H, m, CH_2), 4.08 (1 H, m, CH-OH), 7.26 (1 H, s, CH=N), 7.27 – 7.45 (3 H, m, Ph), 7.85 – 8.25 (2 H, m, Ph).

6ee: δH(500 MHz; $CDCl_3$; Me_4Si) 0.76 (3 H, t, Me), 0.85 – 1.85 (18 H, m, CH_2), 4.44 (1 H, q, CH, CHOH), 4.85 (2 H, s, Bz), 6.68 (1 H, d, CH=N), 7.27 – 7.45 (3 H, m, Ph), 7.85 – 8.25 (5 H, m, Ph).

8a: δH(500 MHz; $CDCl_3$; Me_4Si) 3.44 (2 H, s, CH_2, *cis*-isomer), 3.66 (2 H, s, CH_2, *trans*-isomer), 7.11 – 7.35 (10 H, m, Ph), 8.11 (1 H, br s, NOH).

8b: δH(500 MHz; $CDCl_3$; Me_4Si) 4.28 (4 H, s, CH_2), 7.15 - 755 (10 H, m, Ph), 7.11 – 7.35 (10 H, m, Ph), 9.65 (1 H, br s, NOH).

^{13}C-NMR analysis

4c: δC(125 MHz; $CDCl_3$) 25.50, 56.47, 58.87, 126.92, 128.25, 129.34, 139.79.

4d: δC(125 MHz; $CDCl_3$) 47.61, 66.53, 127.58, 128.35, 129.92, 137.00.

4e: δC(125 MHz; $CDCl_3$) 14.15, 22.73, 25.55, 29.39, 29.70, 31.97, 34.81, 65.39, 65.76, 127.74, 128.45, 129.95, 136.63.

6a: δC(125 MHz; $CDCl_3$) 1.12.57, 13.24, 59.85, 133.55.

6b: δC(125 MHz; $CDCl_3$) 71.32, 128.51, 128.66, 129.01, 129.27, 130.51, 133.36, 134.27.

6c: δC(125 MHz; $CDCl_3$) 28.38, 70.83, 128.45, 128.80, 129.95, 131.11.

6d: δC(125 MHz; $CDCl_3$) 54.41, 128.45, 130.42, 130.55, 135.19.

6e: δC(125 MHz; $CDCl_3$) 14.23, 22.81, 25.52, 29.45, 29.70, 32.04, 34.54, 69.96,70.88, 128.74, 129.17, 130.04, 131.10, 136.42.

6ee: δC(125 MHz; $CDCl_3$) 14.23, 22.81, 25.13, 29.60, 32.03, 33.52, 67.33, 69.42, 129.26, 129.26, 129.43, 129.53, 132.20, 141.78.

8a: δC(125 MHz; $CDCl_3$) 32.71, 39.70, 126.88, 128.67, 129.36, 136.66, 159.13.

8b: δC(125 MHz; $CDCl_3$) 32.73, 126.68, 126.88, 128.91, 129.68, 135.84, 136.73, 157.86.

HRMS (m/z): $[M]^+$

4c: calculated for $C_{11}H_{18}NO$, 180.13884; found 180.13775

4d: calculated for $C_8H_{12}NO$, 138.09189; found 138.09114

4e: calculated for $C_{19}H_{34}NO_2$, 308.25895; found 308.25901

6a: calculated for $C_4H_{10}NO$, 88.07624; found 88.07857

6b: calculated for $C_{14}H_{14}NO$, 212.10754; found 212.10684

6c: calculated for $C_{11}H_{16}NO$, 178.12319; found 178.12222

6d: calculated for $C_{8}H_{10}NO$, 136.07624; found 136.07555

6e: calculated for $C_{19}H_{32}NO$, 306.24330; found 306.24391

6ee: calculated for $C_{19}H_{32}NO$, 306.24330; found 306.24416

8a: calculated for $C_{15}H_{16}NO$, 226.123.19; found 226.12209

8b: calculated for $C_{14}H_{14}NO$, 212.10754; found 212.10679

Copies ^{1}H and ^{13}C NMR spectra (solvent, $CDCl_3$) of hydroxylamines **4b-e** and nitrones **6a-d**.

4c

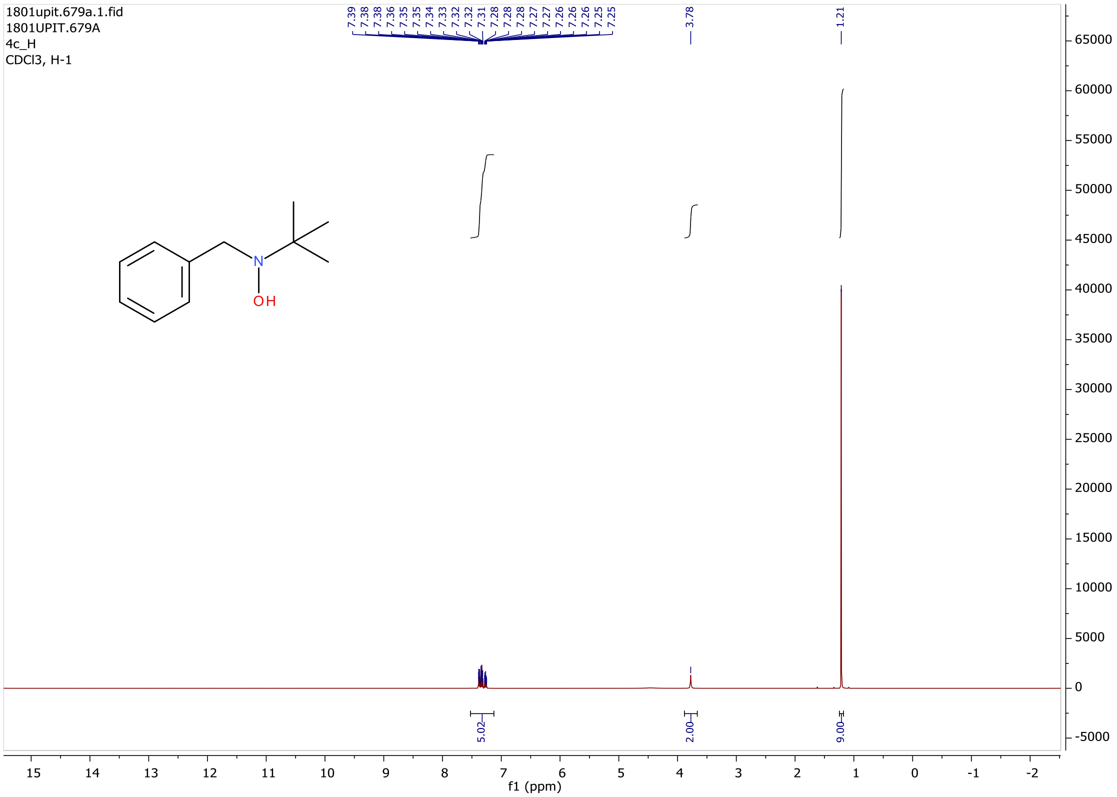

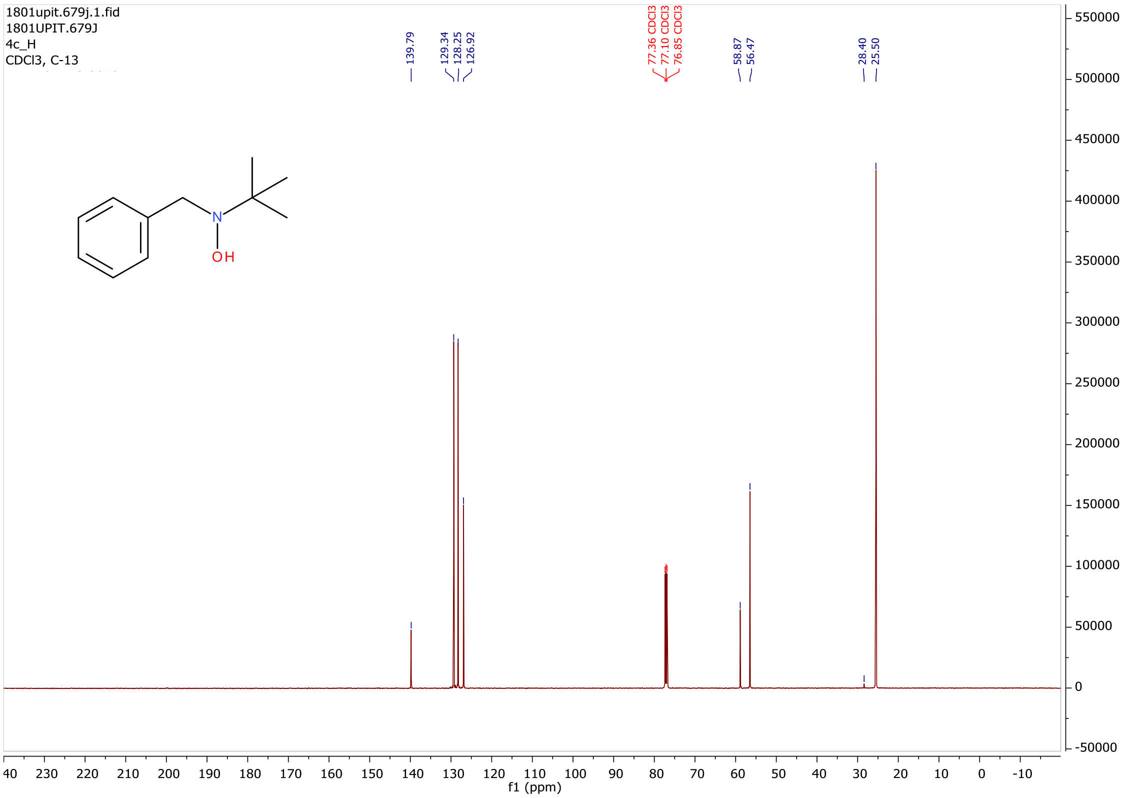
1801upit.679j.1.fid
1801UPIT.679J
4c_H
CDCl3, C-13
139.79
129.34
128.25
126.92
77.36 CDCl3
77.10 CDCl3
76.85 CDCl3
58.87
56.47
28.40
25.50
N
OH
f1 (ppm)

4d

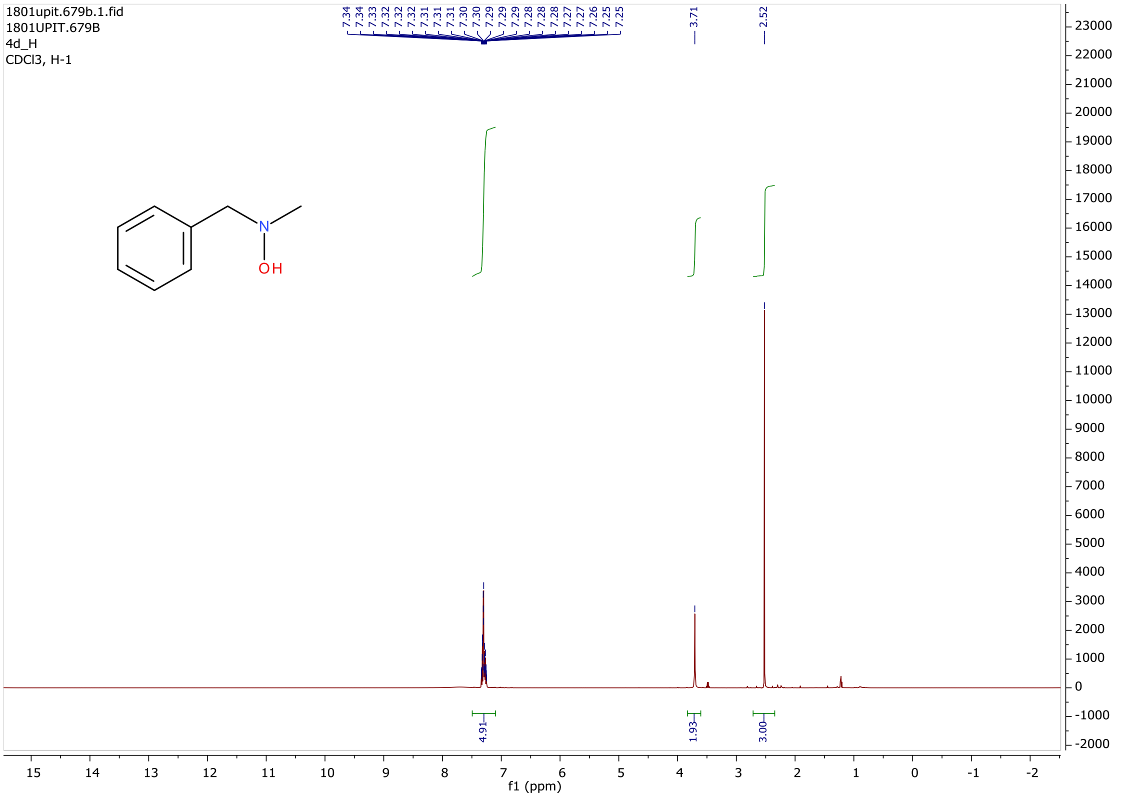
1801upit.679b.1.fid
1801UPIT.679B
4d_H
CDCl3, H-1
7.34
7.34
7.33
7.32
7.32
7.32
7.31
7.31
7.30
7.30
7.29
7.29
7.29
7.28
7.28
7.28
7.27
7.27
7.26
7.25
7.25
3.71
2.52
N
OH
4.91
1.93
3.00
f1 (ppm)

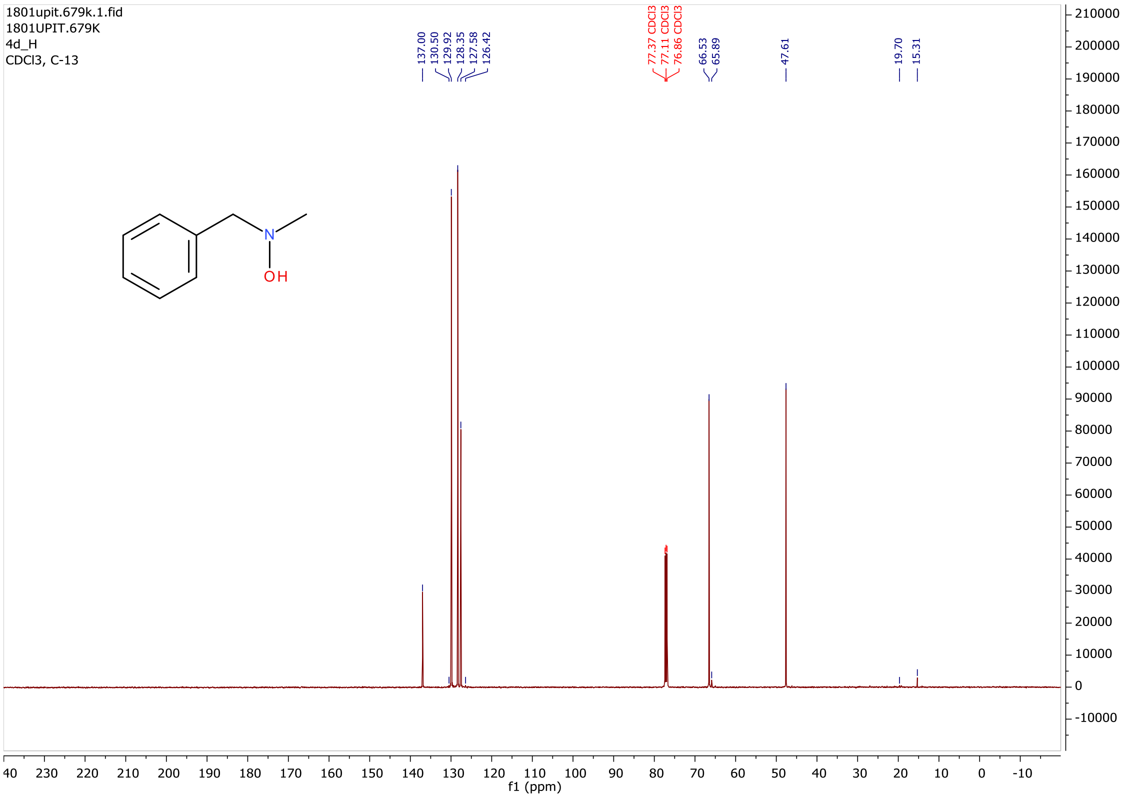
1801upit.679k.1.fid
1801UPIT.679K
4d_H
CDCl3, C-13
137.00
130.50
129.92
128.35
127.58
126.42
77.37 CDCl3
77.11 CDCl3
76.86 CDCl3
66.53
65.89
47.61
19.70
15.31
N
OH
f1 (ppm)

6a

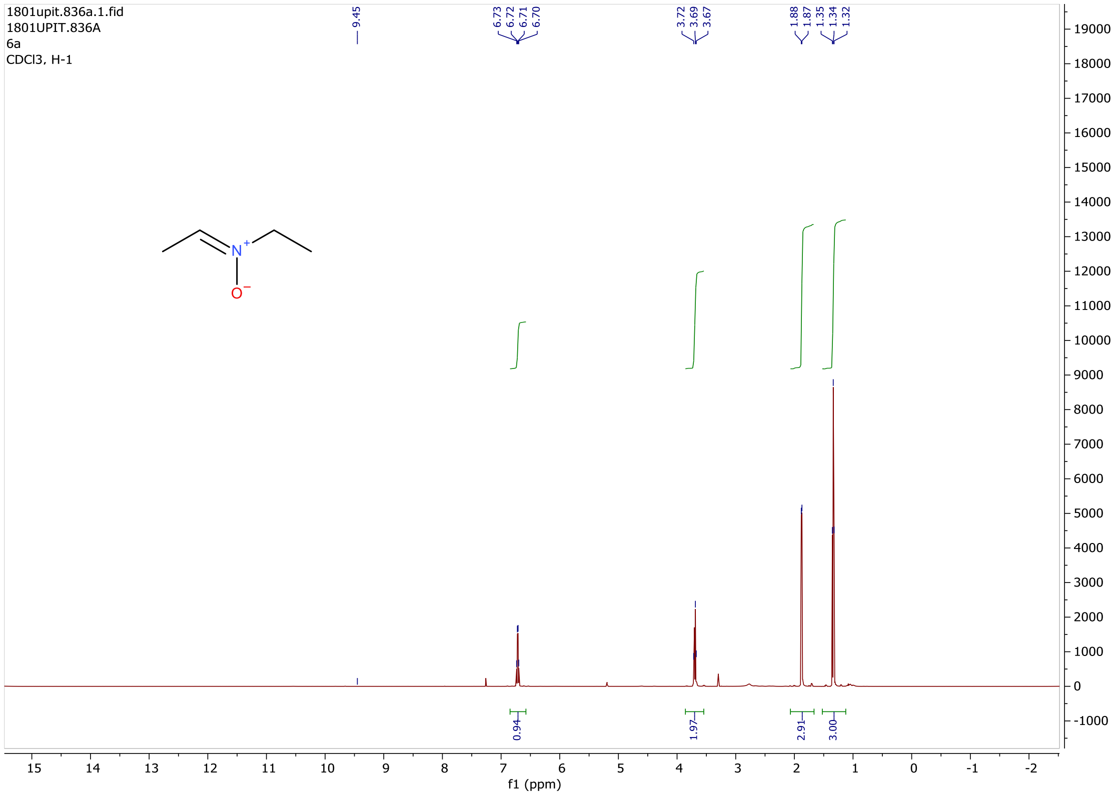
1801upit.836a.1.fid
1801UPIT.836A
6a
CDCl3, H-1
9.45
6.73
6.72
6.71
6.70
3.72
3.69
3.67
1.88
1.87
1.35
1.34
1.32
N+
O−
0.94
1.97
2.91
3.00
f1 (ppm)

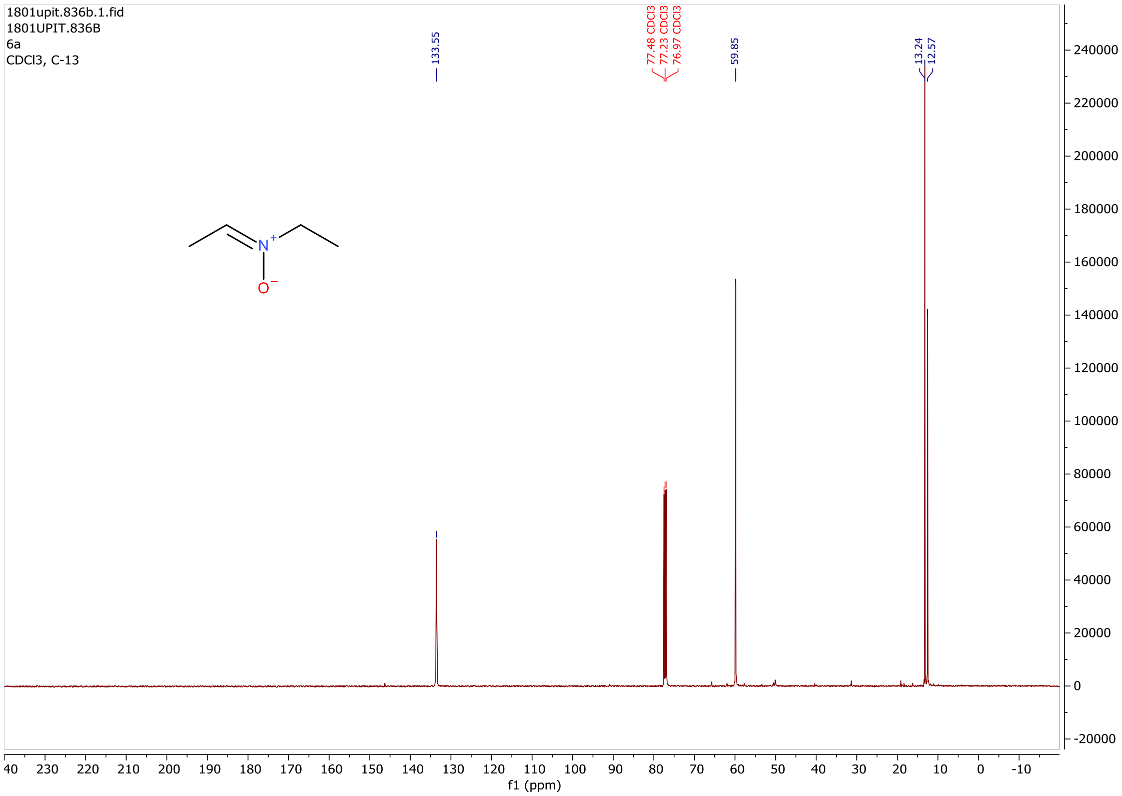
1801upit.836b.1.fid
1801UPIT.836B
6a
CDCl3, C-13
133.55
77.48 CDCl3
77.23 CDCl3
76.97 CDCl3
59.85
13.24
12.57
f1 (ppm)

6b

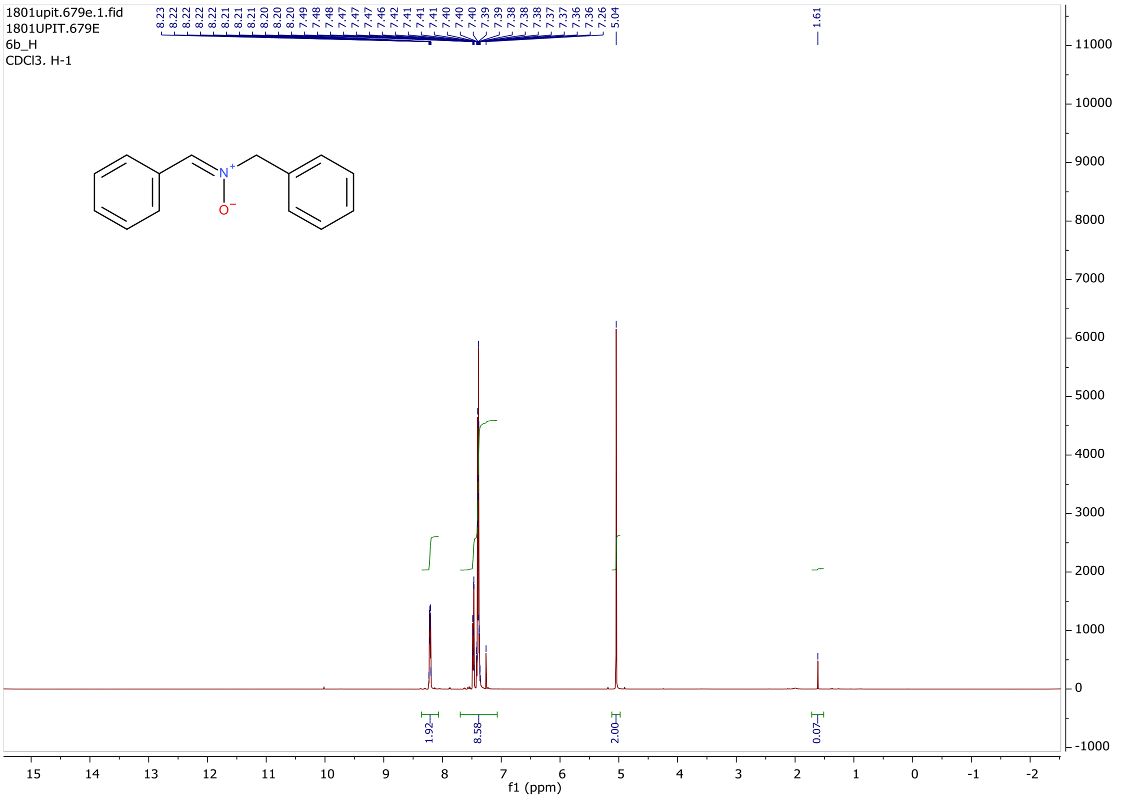
1801upit.679e.1.fid
1801UPIT.679E
6b_H
CDCl3. H-1
8.23 8.22 8.22 8.22 8.22 8.21 8.21 8.21 8.20 8.20 8.20 7.49 7.48 7.48 7.47 7.47 7.47 7.46 7.42 7.41 7.41 7.41 7.40 7.40 7.40 7.39 7.39 7.38 7.38 7.38 7.37 7.37 7.36 7.36 7.26
5.04
1.61
1.92
8.58
2.00
0.07
f1 (ppm)

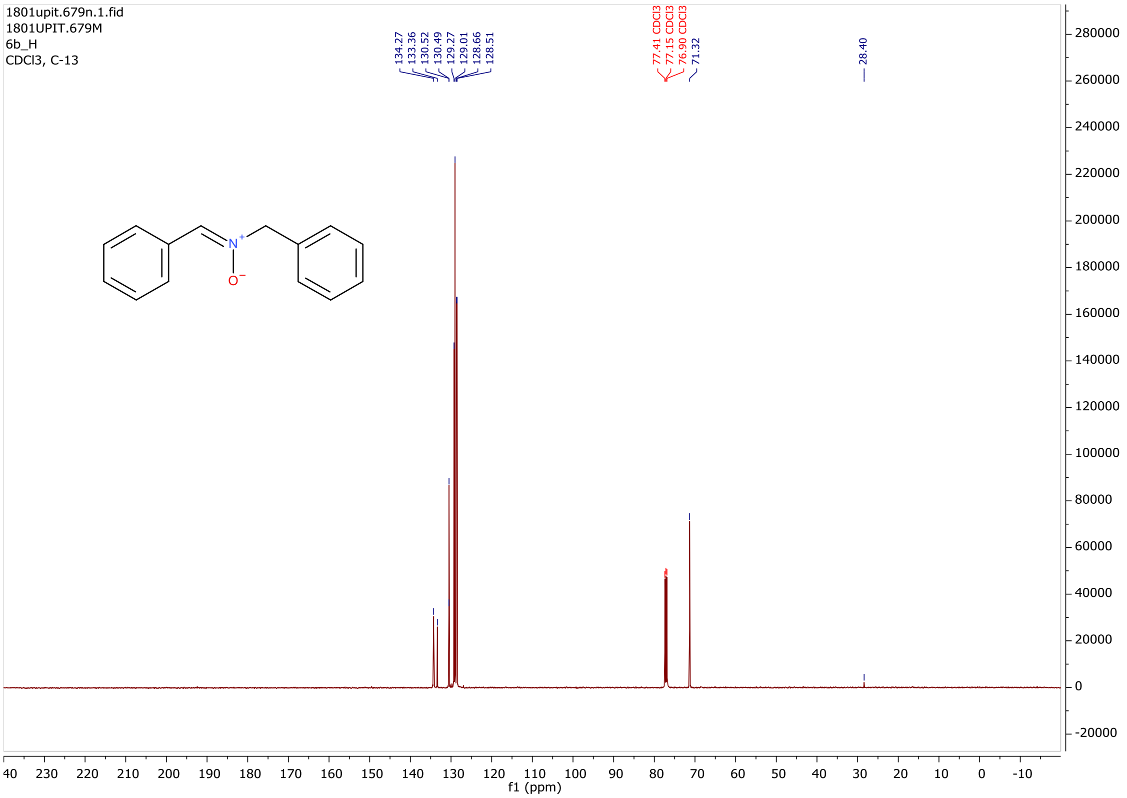
1801upit.679n.1.fid
1801UPIT.679M
6b_H
CDCl3, C-13
134.27
133.36
130.52
130.49
129.27
129.01
128.66
128.51
77.41 CDCl3
77.15 CDCl3
76.90 CDCl3
71.32
28.40
f1 (ppm)

6c

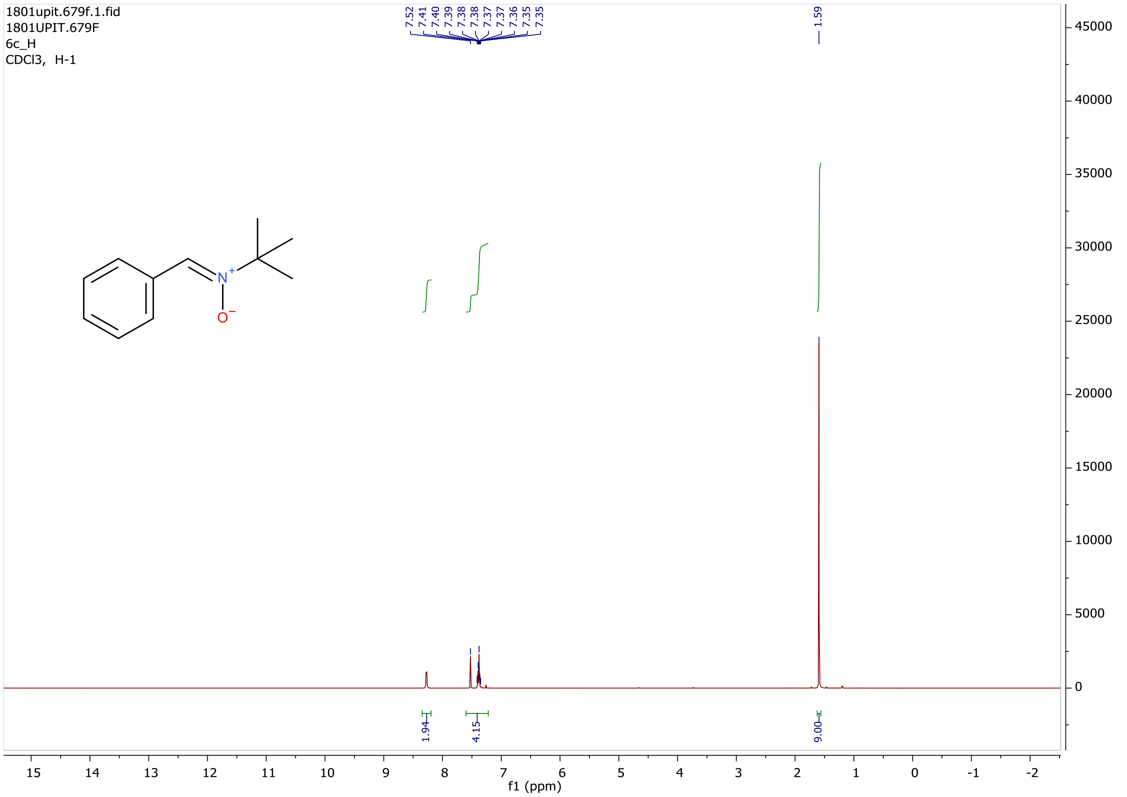
1801upit.679f.1.fid
1801UPIT.679F
6c_H
CDCl3, H-1
7.52
7.41
7.40
7.39
7.38
7.38
7.37
7.36
7.35
7.35
1.59
1.94
4.15
9.00
f1 (ppm)

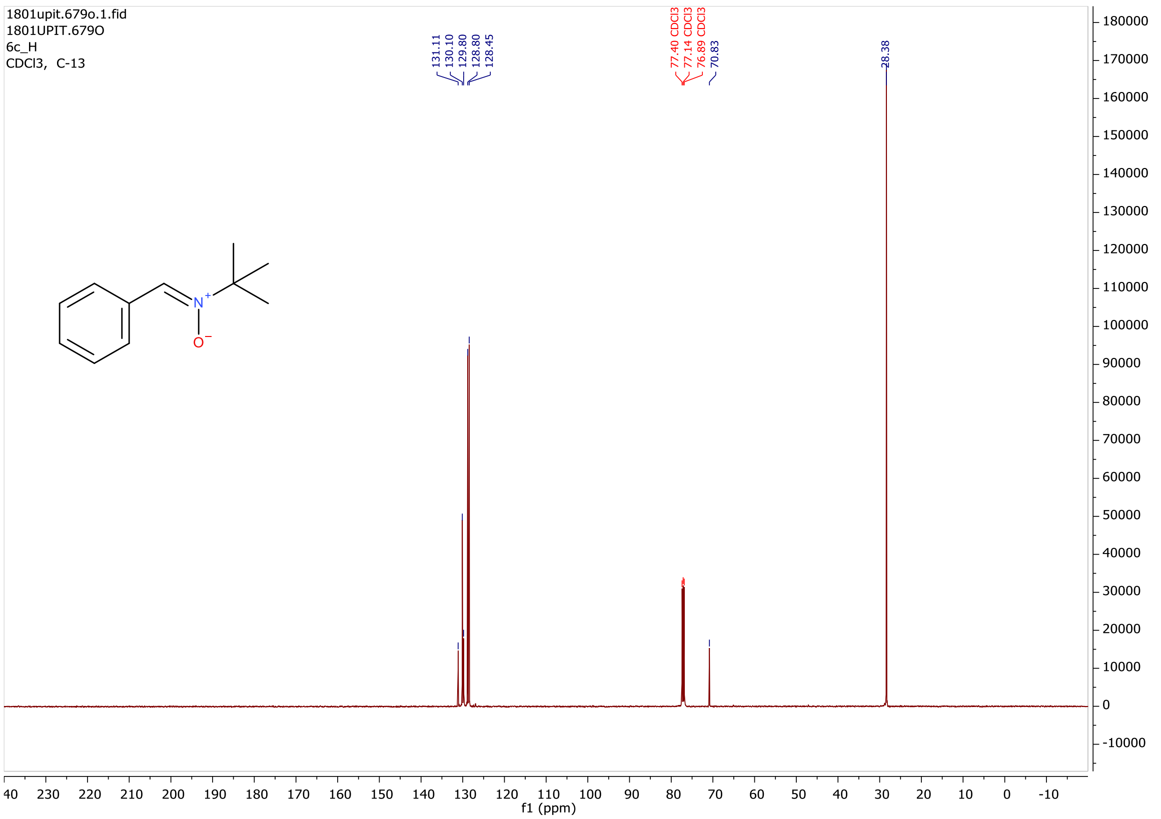
1801upit.679o.1.fid
1801UPIT.679O
6c_H
CDCl3, C-13
131.11
130.10
129.80
128.80
128.45
77.40 CDCl3
77.14 CDCl3
76.89 CDCl3
70.83
28.38
f1 (ppm)

6d

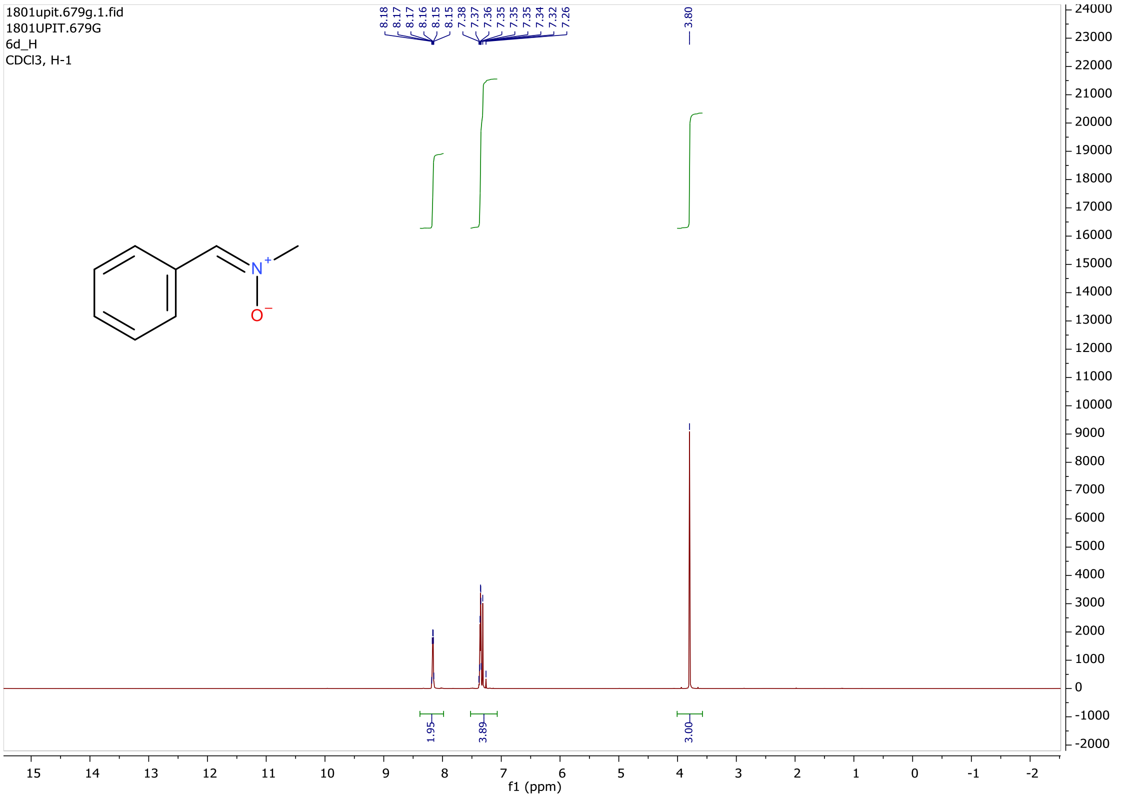
1801upit.679g.1.fid
1801UPIT.679G
6d_H
CDCl3, H-1
8.18
8.17
8.17
8.16
8.15
8.15
7.38
7.37
7.36
7.35
7.35
7.35
7.34
7.32
7.26
3.80
1.95
3.89
3.00
f1 (ppm)

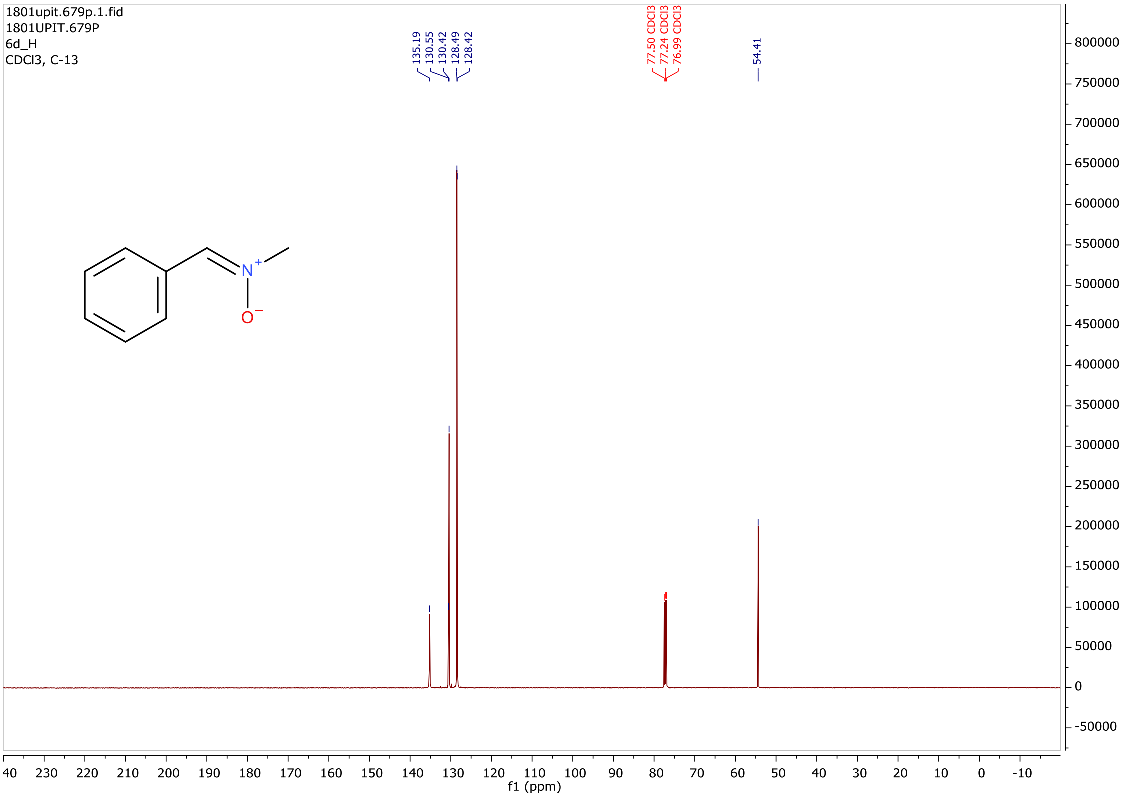
1801upit.679p.1.fid
1801UPIT.679P
6d_H
CDCl3, C-13
135.19
130.55
130.42
128.49
128.42
77.50 CDCl3
77.24 CDCl3
76.99 CDCl3
54.41
f1 (ppm)

8a

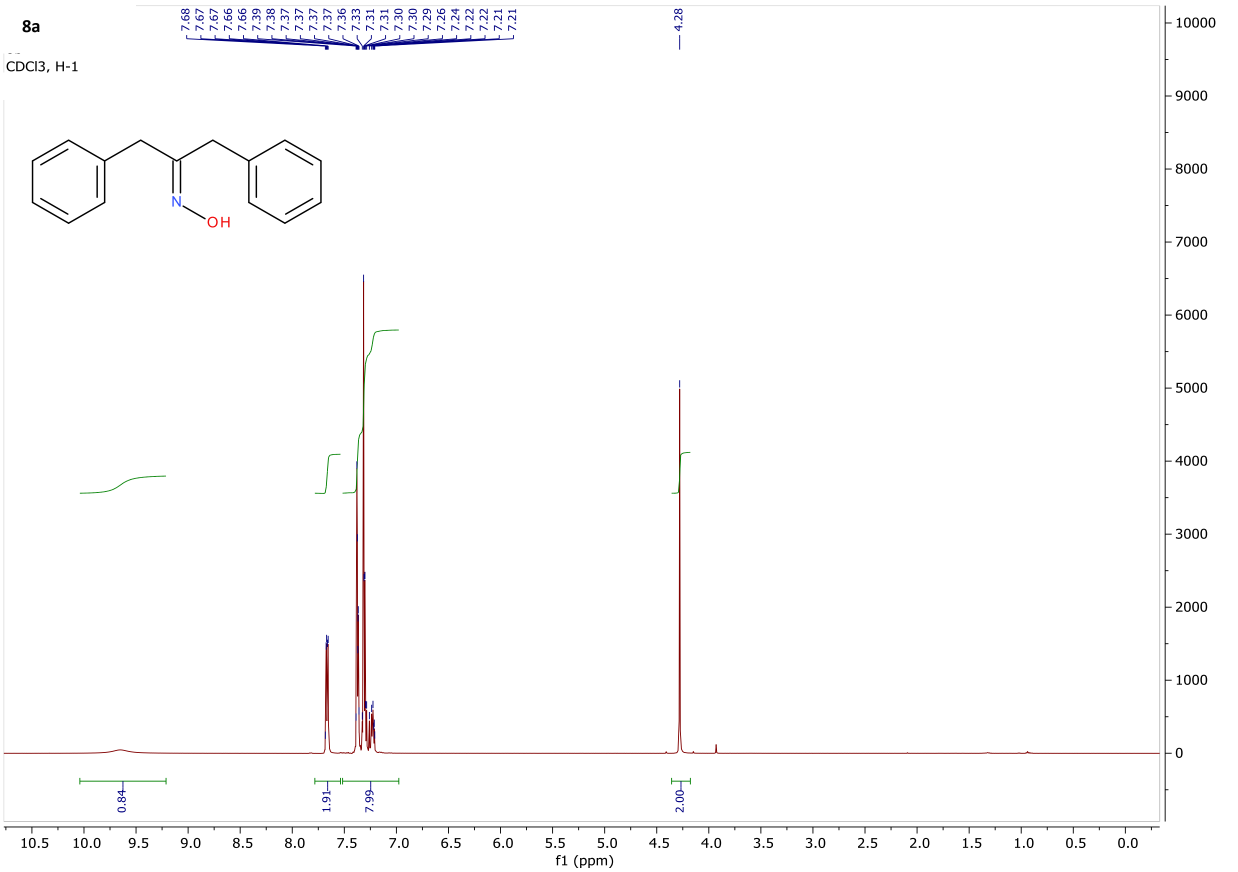

8a
CDCl3, H-1
7.68 7.67 7.67 7.66 7.66 7.39 7.38 7.37 7.37 7.37 7.37 7.36 7.33 7.31 7.31 7.30 7.30 7.29 7.26 7.24 7.22 7.22 7.21 7.21
4.28
N
OH
0.84
1.91
7.99
2.00
f1 (ppm)

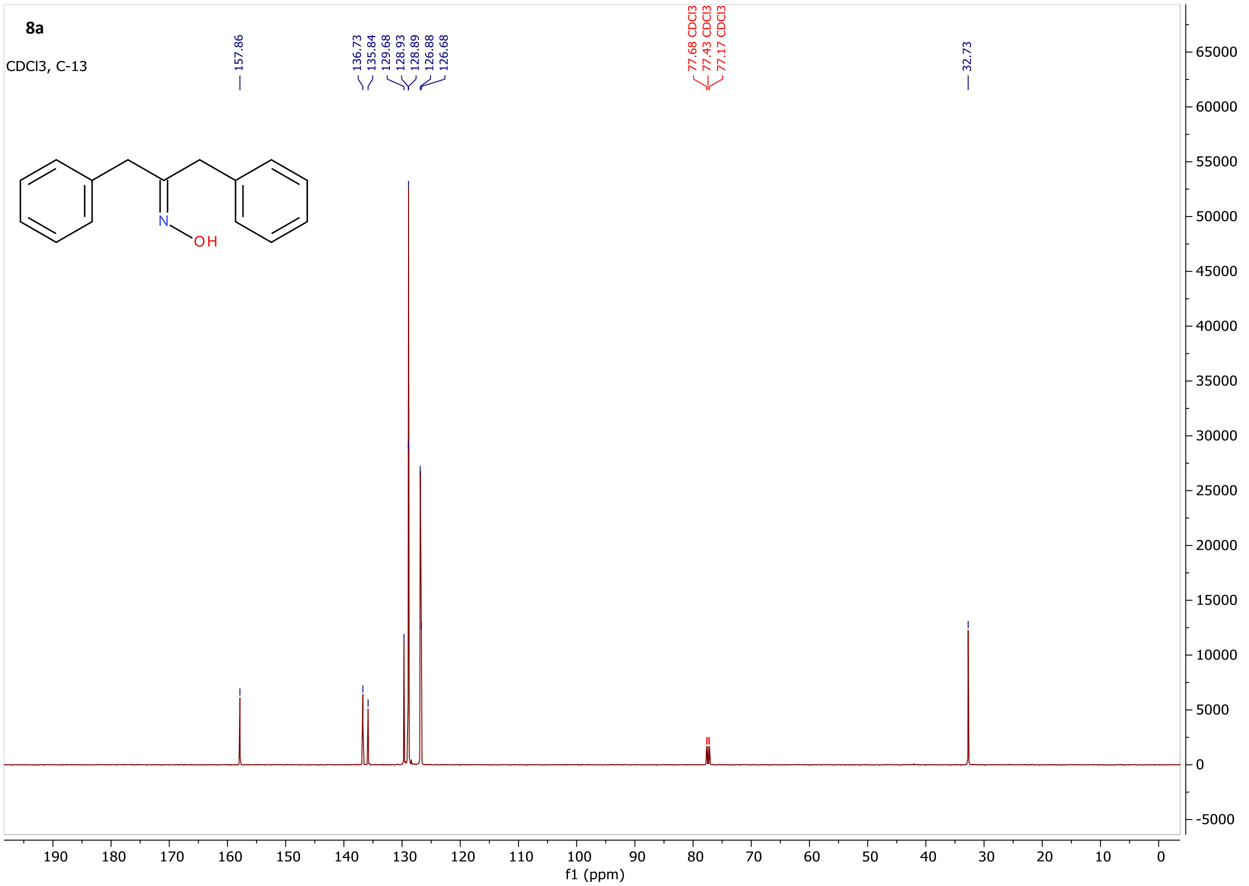

8a
CDCl3, C-13
157.86
136.73 135.84 129.68 128.93 128.89 126.88 126.68
77.68 CDCl3 77.43 CDCl3 77.17 CDCl3
32.73
N
OH
f1 (ppm)

8b

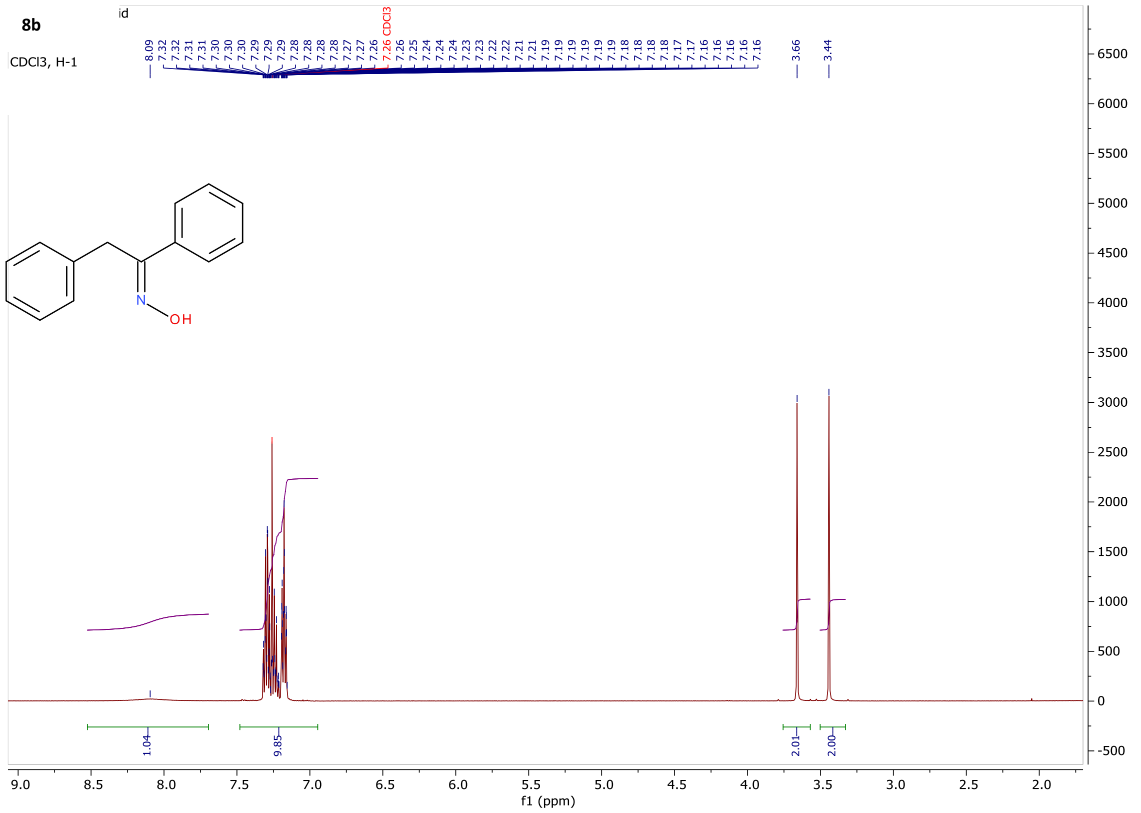

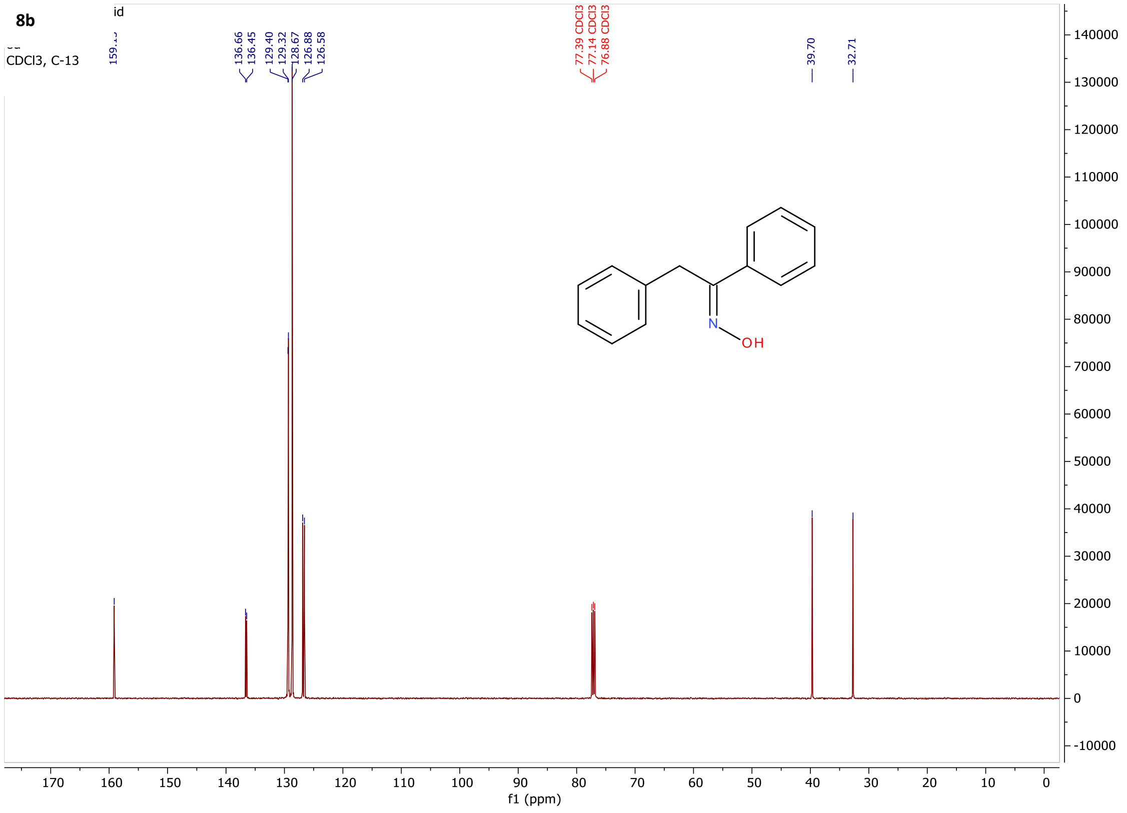

Figure 1S. HPLC separation of nitrones **6e** and **6ee**. A- Crude reaction product (50 mg), isolated after oxidation of **4e** with **1.Br**, was dissolved in ethanol (90%; 4 mL) and subjected to HPLC separation on a C18 column (2.12 x 25 cm; 5 μm particle size; Supelco, Inc.; Bellefonte, PA). The analytes were eluted with 70% methanol for 50 min and then with 100% methanol for 70 min at a flow rate of 5 mL/min. Inset- UV spectra of the compounds eluted under peaks 1 and 2 (λ_{max} = 232 nm and 290 nm, respectively). B- Contour map of the HPLC separation in the wavelength range of 200 to 400 nm.

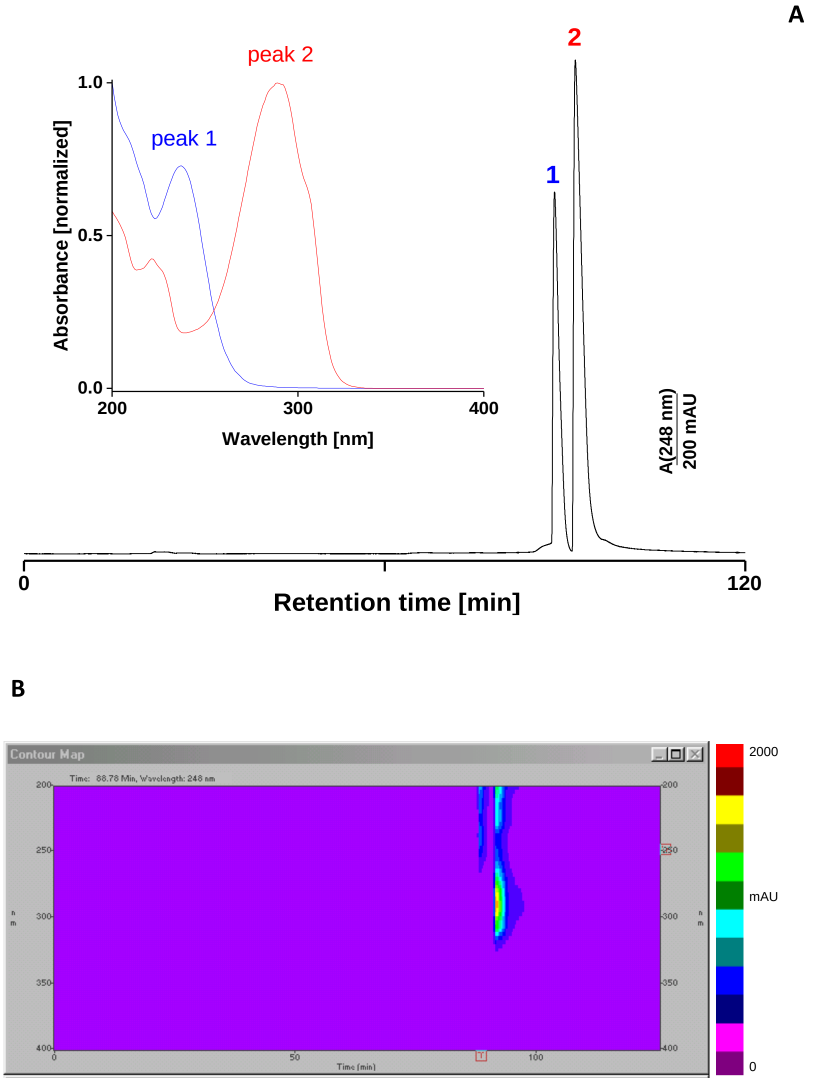

Figure 2S. HRMS, MS/MS and NMR analysis of nitrones **6ee** and **6e** (Fig. 1S, analytes eluted under peaks 1 and 2).

Peak 1, MS (6ee)

79289ESIPMStMS1#38-64 RT: 0.14-0.24 AV: 14
T: FTMS + p ESI Full ms [100.0000-1000.0000]
m/z= 306.00000-307.00000

m/z	Intensity	Relative	Theo. Mass	Delta (mmu)	Compos.
306.24416	2770233344.0	100.00	306.24276	1.41	$C_{19}H_{32}O_2$

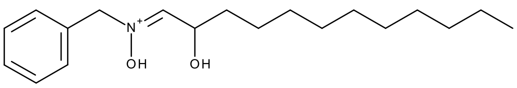

Protonated form of **6ee** - exact mass, 306.24330
Molecular Formula: $C_{19}H_{32}NO_2$

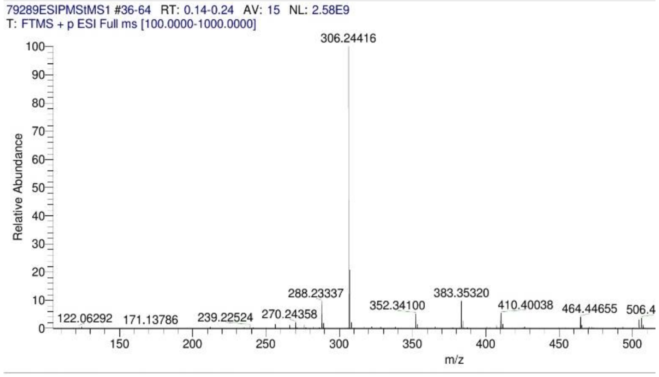

Peak 2, MS (6e)

79288esipmstms1#54-90 RT: 0.20-0.34 AV: 19
T: FTMS + p ESI Full ms [100.0000-1000.0000]
m/z= 50.00000-1000.00000

m/z	Intensity	Relative	Theo. Mass	Delta (ppm)	Comp
306.24390	7229043712.0	100.00	306.24276	1.15	$C_{19}H_{32}$

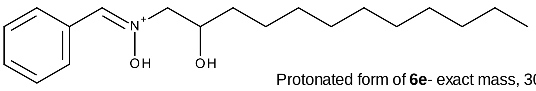

Protonated form of **6e**- exact mass, 306.24330
Molecular Formula: $C_{19}H_{32}NO_2$

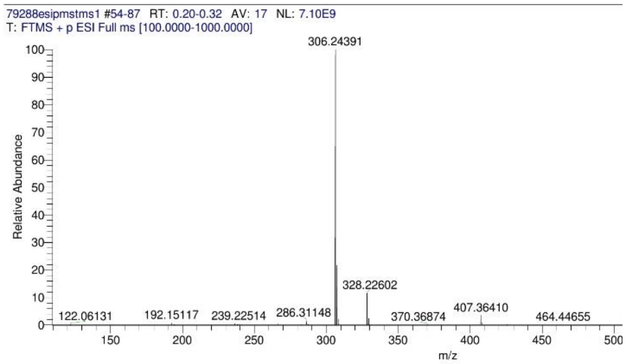

Peak 1, MS/MS (parent ion, m/z = 306)

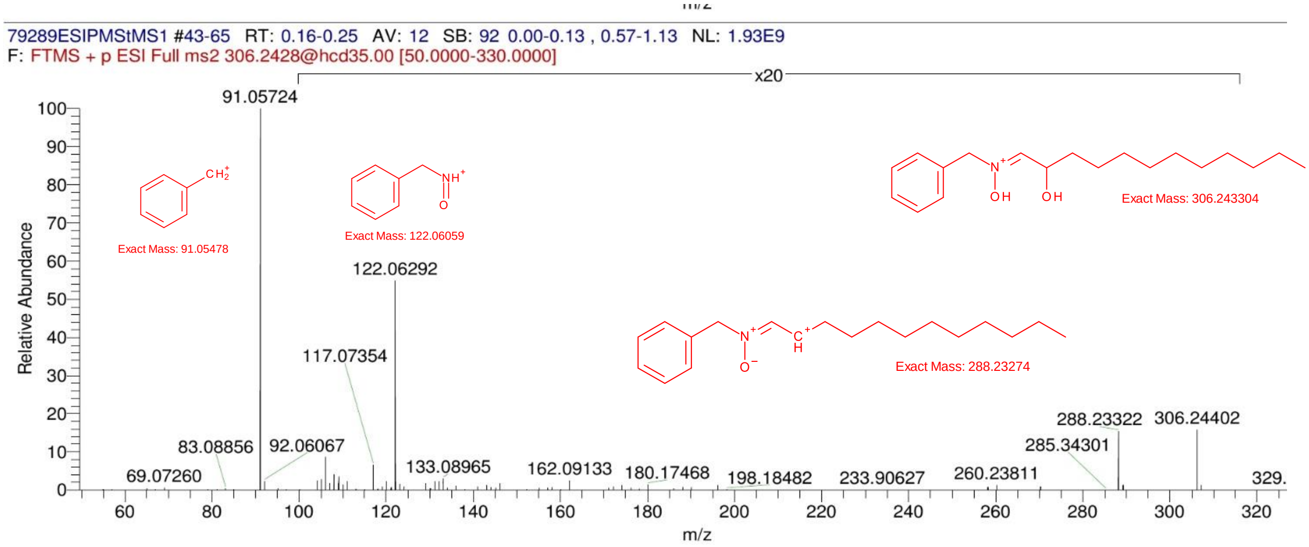

Peak 2, MS/zMS (parent ion, m/z = 306)

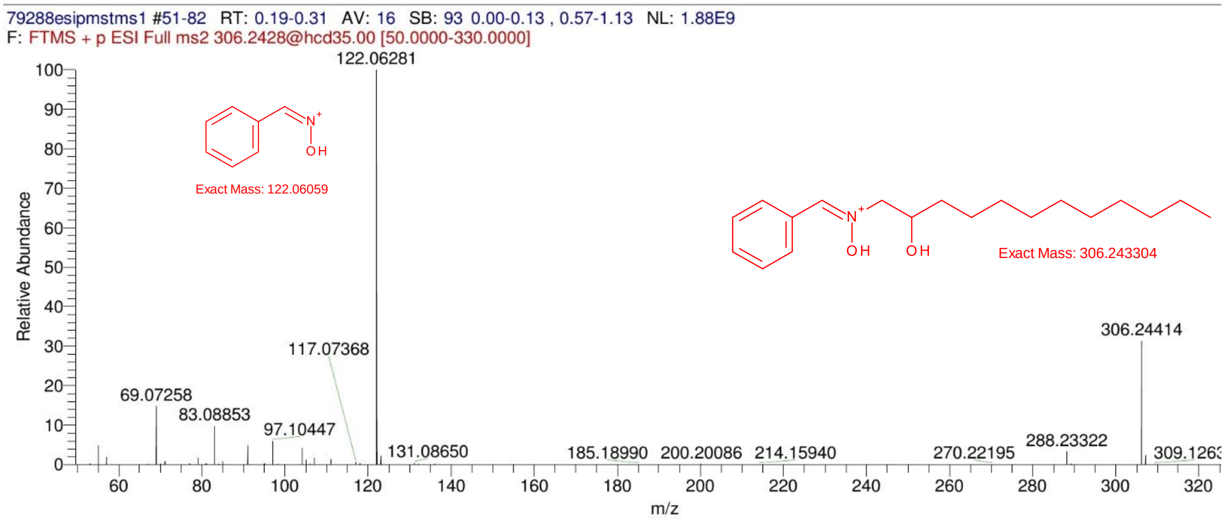

Copies of NMR spectra of nitrone **6ee** (Fig. 1SA, compound eluted under peak 1).

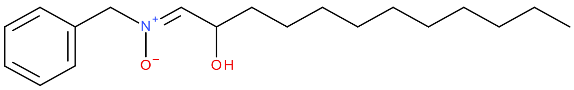

^{1}H NMR (solvent, $CDCl_3$)

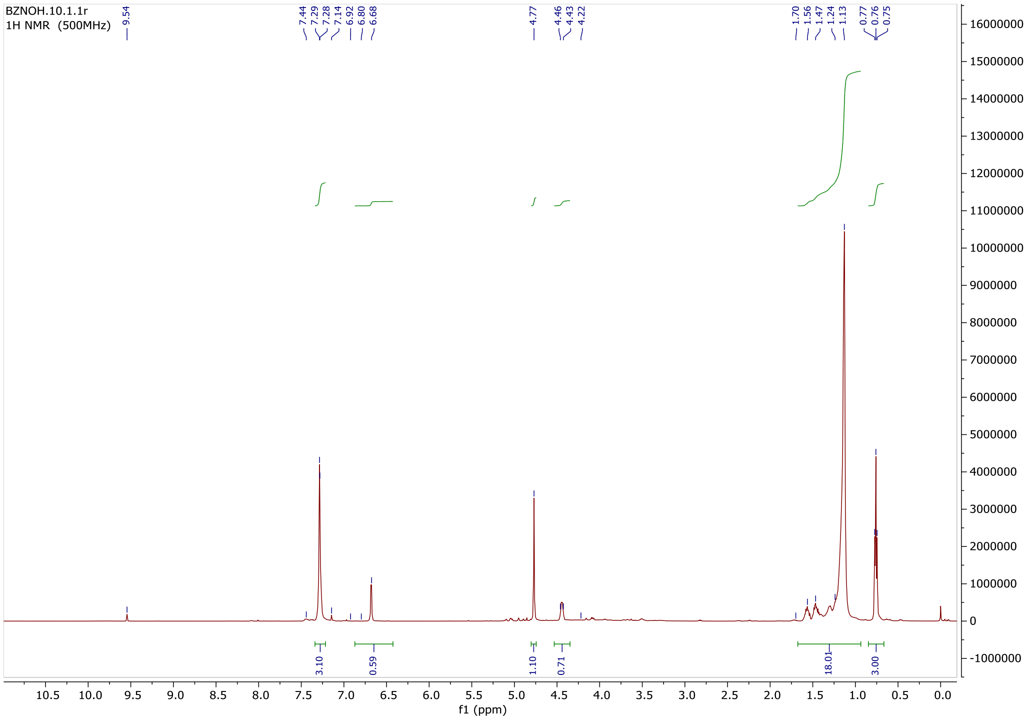

^{13}C NMR (solvent, $CDCl_3$)

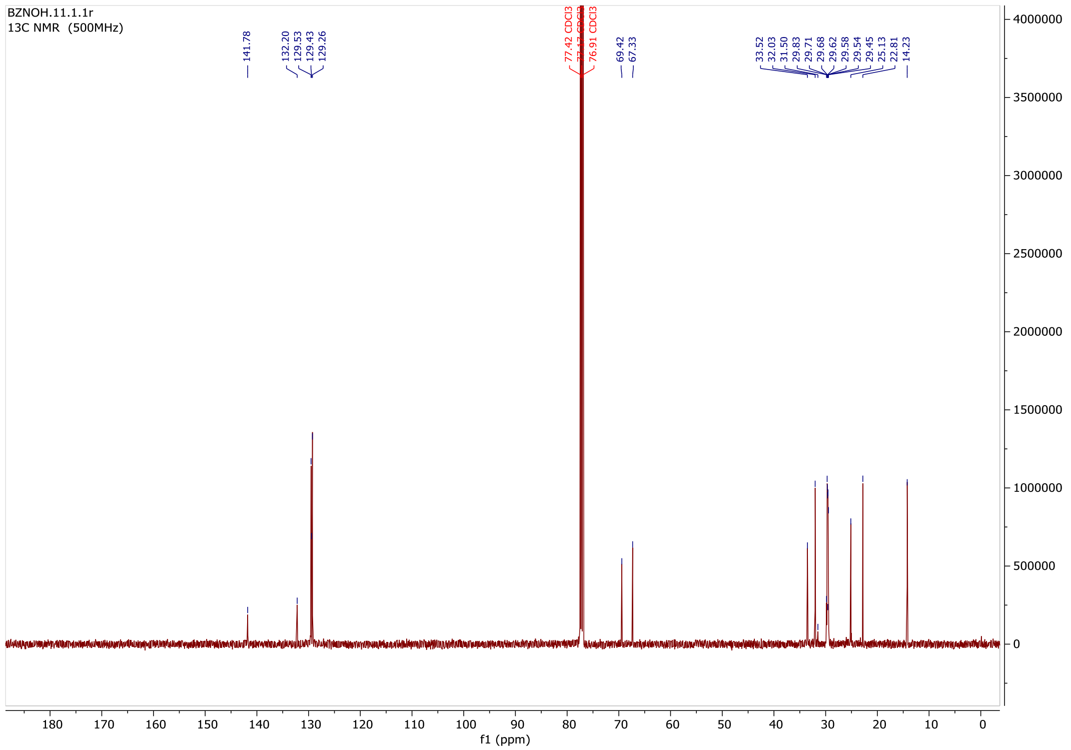

Copies of NMR spectra of nitrone **6e** (Fig. 1S, compound eluted under peak 2).

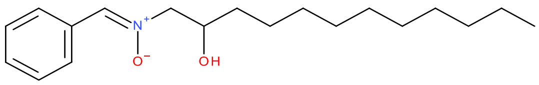

^{1}H NMR (solvent, $CDCl_3$)

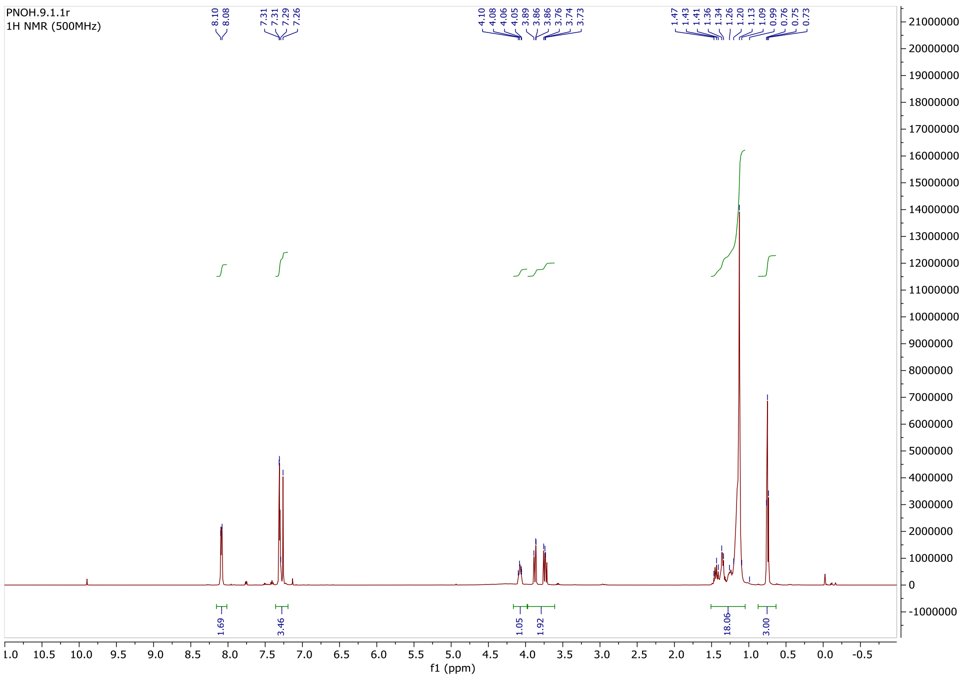

^{13}C NMR (solvent, $CDCl_3$)

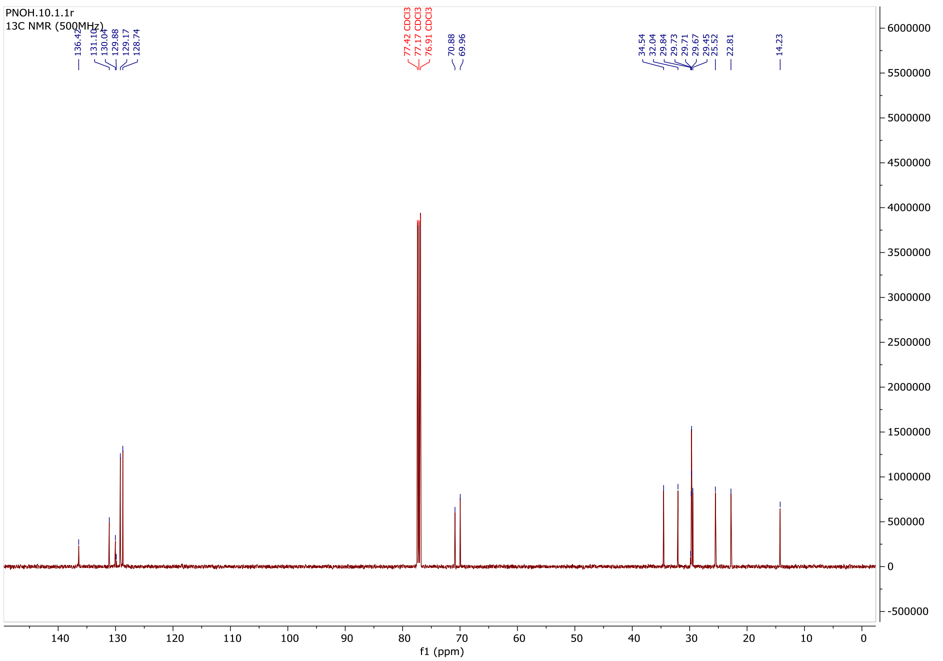

Copies of HRMS spectra of hydroxylamines **4b-e**, nitrones **6a-d**, and oximes **8a,b**.

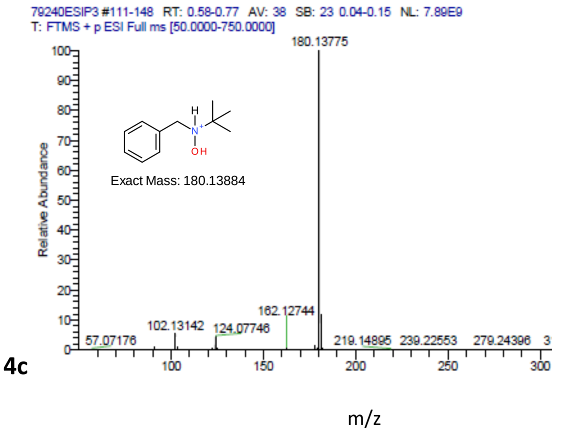

4c

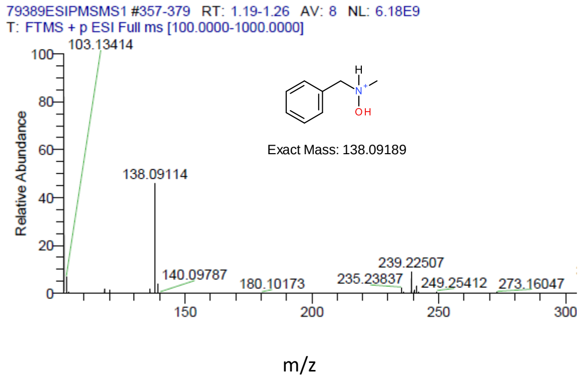

4d

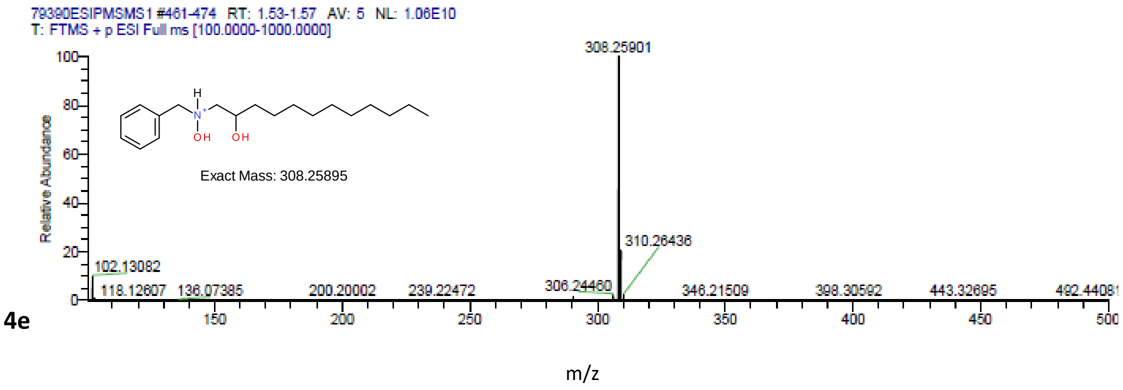

4e

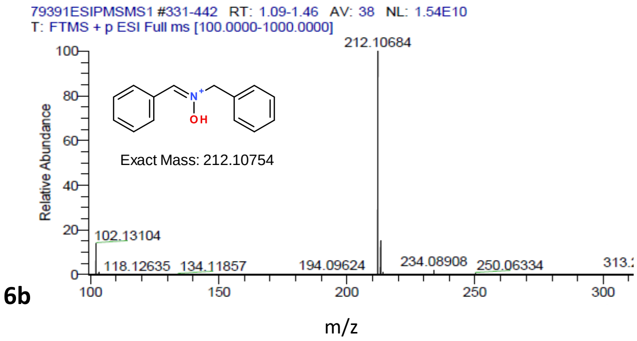

6b

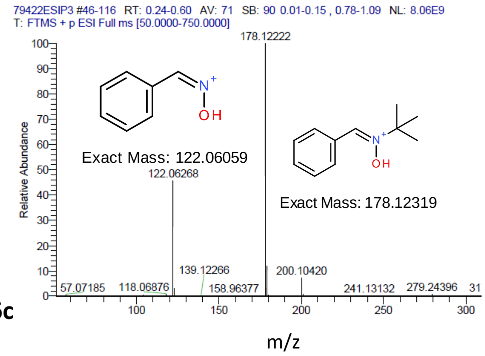

6c

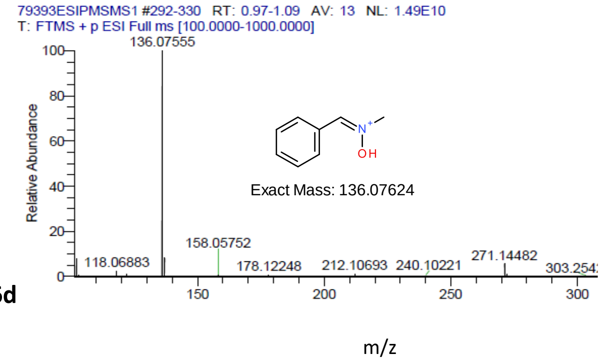

6d

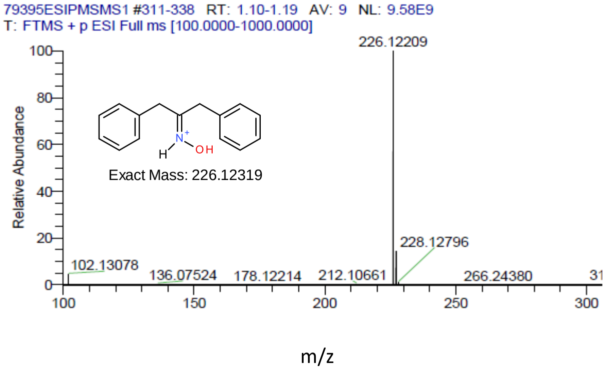

8a

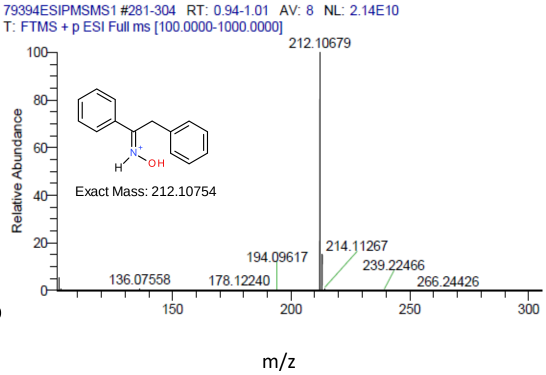

8b

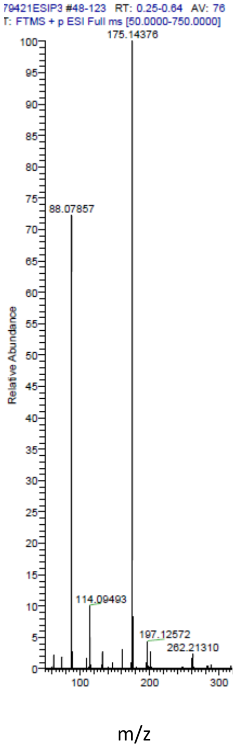

6a

Scheme 1

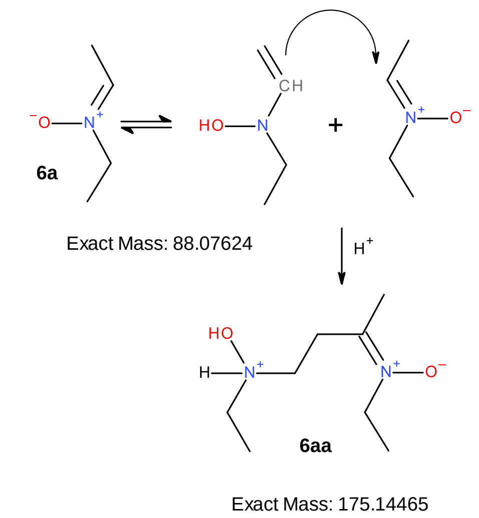

Nitrone **6a** readily dimerizes to hydroxylamine **6aa** (Scheme 1; ESI source, 150 °C).[6]

Figure 3S. HPLC-UV chromatograms of nitrones **6a-d** (0.3 mM; blue tracings; panels A-B) obtained via oxidation of hydroxylamine **4a-d** by **1.Br**. Red tracings- HPLC chromatograms of authentic nitrones used as external reference standards. Insets- UV spectra of the compounds eluted under the peaks of synthetic (blue tracings) and reference nitrones (red tracings).

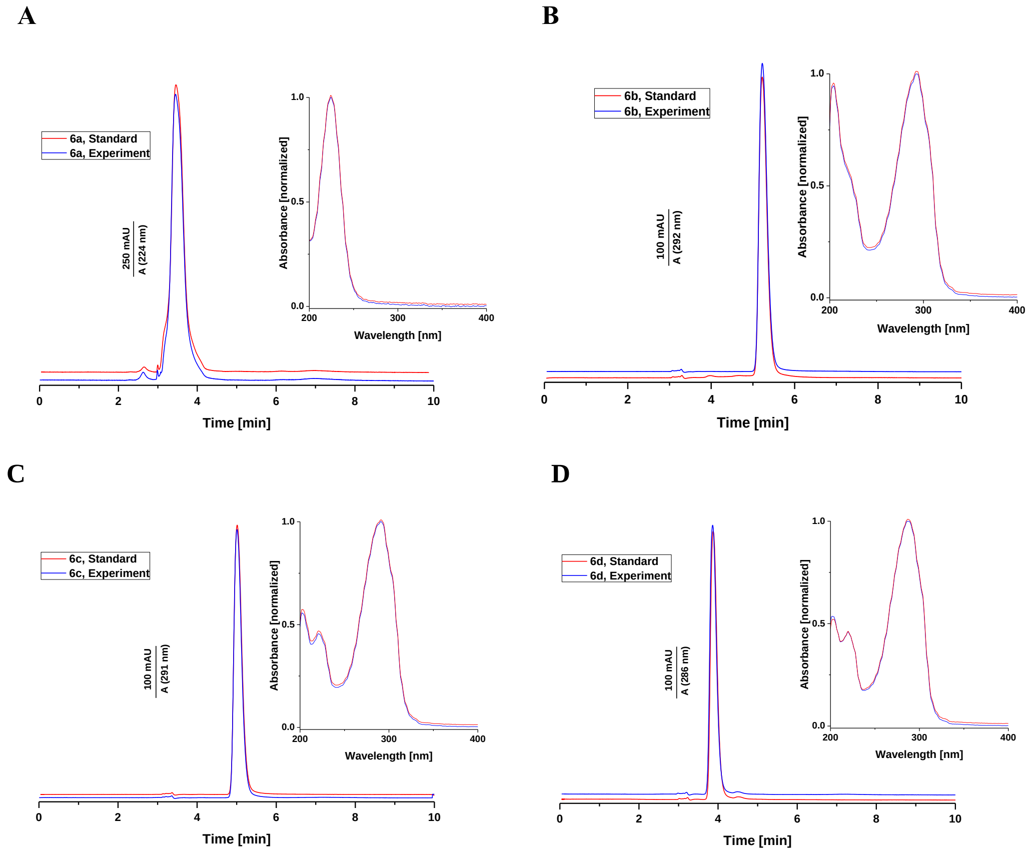

References

[1] H. F. Schmitthenner, K. S. Bhatki, R. A. Olofson, J. Heicklen, *Org. Prep. Proced. Int.* **1979**, *11*, 249-251.

[2] H. X. Ding, S. G. Hong, N. Zhang, *Tetrahedron Lett.* **2015**, *56*, 507-510.

[3] S. Colonna, V. Pironti, G. Carrea, P. Pasta, F. Zambianchi, *Tetrahedron* **2004**, *60*, 569-575.

[4] I. A. O'Neil, J. M. Southern, *Tetrahedron Lett.* **1998**, *39*, 9089-9092.

[5] N. Merbouh, J. M. Bobbitt, C. Bruckner, *Org. Prep. Proced. Int.* **2004**, *36*, 1-31.

[6] D. Barasch, M. C. Krishna, A. Russo, J. Katzhendler, A. Samuni, *J. Am. Chem. Soc.* **1994**, *116*, 7319-7324.
